# Combining spaceborne lidar from the Global Ecosystem Dynamics Investigation with local knowledge for monitoring fragmented tropical landscapes: A case study in the forest–agriculture interface of Ucayali, Peru

**DOI:** 10.1002/ece3.70116

**Published:** 2024-08-06

**Authors:** Savannah S. Cooley, Naiara Pinto, Milagros Becerra, Jorge Washington Vela Alvarado, Jocelyn C. Fahlen, Ovidio Rivera, G. Andrew Fricker, Augusto Rafael De Los Ríos Dantas, Naikoa Aguilar‐Amuchastegui, Yunuen Reygadas, Julie Gan, Ruth DeFries, Duncan N. L. Menge

**Affiliations:** ^1^ NASA Jet Propulsion Laboratory California Institute of Technology Pasadena California USA; ^2^ Department of Ecology, Evolution, and Environmental Biology Columbia University New York New York USA; ^3^ Conservación Amazónica Lima Peru; ^4^ Universidad Nacional de Ucayali Pucallpa Peru; ^5^ International Center for Tropical Agriculture Cali Colombia; ^6^ Department of Social Sciences California Polytechnic State University San Luis Obispo California USA; ^7^ Dirección de Gestión del Territorio de la Autoridad Regional Ambiental del Gobierno Regional de Ucayali Pucallpa Peru; ^8^ The World Wildlife Fund, Forest and Climate Program Washington DC USA; ^9^ Department of Geosciences Texas Tech University Lubbock Texas USA

**Keywords:** co‐production, ecological succession, forest structure, local knowledge, spaceborne lidar, tropical lowland forest, Ucayali, Peru

## Abstract

Improving our ability to monitor fragmented tropical ecosystems is a critical step in supporting the stewardship of these complex landscapes. We investigated the structural characteristics of vegetation classes in Ucayali, Peru, employing a co‐production approach. The vegetation classes included three agricultural classes (mature oil palm, monocrop cacao, and agroforestry cacao plantations) and three forest regeneration classes (mature lowland forest, secondary lowland forest, and young lowland vegetation regrowth). We combined local knowledge with spaceborne lidar from NASA's Global Ecosystem Dynamics Investigation mission to classify vegetation and characterize the horizontal and vertical structure of each vegetation class. Mature lowland forest had consistently higher mean canopy height and lower canopy height variance than secondary lowland forest (μ = 29.40 m, sd = 6.89 m vs. μ = 20.82 m, sd = 9.15 m, respectively). The lower variance of mature forest could be attributed to the range of forest development ages in the secondary forest patches. However, secondary forests exhibited a similar vertical profile to mature forests, with each cumulative energy percentile increasing at similar rates. We also observed similar mean and standard deviations in relative height ratios (RH50/RH95) for mature forest, secondary forest, and oil palm even when removing the negative values from the relative height ratios and interpolating from above‐ground returns only (mean RH50/RH95 of 0.58, 0.54, and 0.53 for mature forest, secondary forest, and oil palm, respectively) (*p* < .0001). This pattern differed from our original expectations based on local knowledge and existing tropical forest succession studies, pointing to opportunities for future work. Our findings suggest that lidar‐based relative height metrics can complement local information and other remote sensing approaches that rely on optical imagery, which are limited by extensive cloud cover in the tropics. We show that characterizing ecosystem structure with a co‐production approach can support addressing both the technical and social challenges of monitoring and managing fragmented tropical landscapes.

## Graphical abstract

We employed a co‐production approach leveraging spaceborne lidar and local knowledge to investigate the structural characteristics of the forest‐agriculture interface in Ucayali, Peru. Results indicated higher relative height ratios (RH50/RH95 and RH75/RH95) for mature forest compared to secondary forest (*p* < .0001), even after accounting for the influence of total canopy height on the relative height ratios. This pattern differed from our original expectations based on local knowledge and existing tropical forest succession studies, pointing to numerous opportunities for future work.

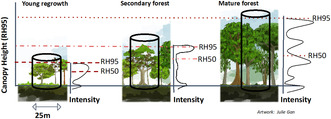

## 1 INTRODUCTION

### 1.1 Monitoring fragmented tropical landscapes

Fragmented forested landscapes are increasingly prevalent due to ongoing deforestation and land‐use change, with the highest rates of fragmentation occurring in the tropics (Taubert et al., 2018). Numerous technical and socio‐ecological challenges arise in monitoring and stewarding fragmented tropical landscapes. Cycles of tropical forest fragmentation, degradation, deforestation, and regeneration influence trajectories of carbon accumulation (from biomass), biodiversity, and human livelihood activity (Arroyo‐Rodríguez et al., 2017; Chazdon, 2014; Peña‐Claros, 2003). Tropical forests generally have higher biodiversity relative to temperate and boreal forests (Mittelbach et al., 2007; Ogar et al., 2020). In contrast to slower‐growing temperate or boreal forests, tropical forests are highly resilient to low‐intensity land use: after 20 years, 12 distinct forest attributes were found to attain 78% (33–100%) of their old‐growth values (Poorter et al., 2021). However, among these attributes, recovery to 90% of old‐growth values was slowest for biomass and species composition (>12 decades) and intermediate for structure and species diversity (2.5–6 decades) (Poorter et al., 2021). Fragmentation and other land use disturbances can significantly alter biomass accumulation and biodiversity outcomes—leading to large variability and associated technical challenges in modeling tropical forest structure, carbon, and biodiversity recovery (Cook‐Patton et al., 2020; Li & Jiang, 2021; Norden et al., 2015; Pugh et al., 2019).

Socioecological challenges emerge alongside the aforementioned technical challenges of monitoring and stewarding fragmented tropical landscapes. These include the disconnect of short‐term thinking in global and national forest governance planning versus the long timescales of biodiversity and carbon recovery (Anderson‐Teixeira et al., 2013; Chazdon et al., 2007; Ogar et al., 2020; Raczka et al., 2023) as well as the balance of seemingly conflicting land use priorities such as livelihood support, climate mitigation, and biodiversity protection (Crouzeilles et al., 2020; Nelson et al., 2009). Wide‐spread reversals in reforestation, leakage effects, and forest degradation further challenge the effectiveness of existing international and national conservation efforts (Schleicher et al., 2017; Schwartz et al., 2020). Yet these technical and governance complexities of monitoring and managing fragmented tropical ecosystems are often centered around distinct technical measurements, thus sharing a similar onto‐epistemological world of forest defining and singularization (Turnhout & Lynch, 2024) and resulting in a major limitation in their adaptability and relevance to local experiences and practices (Myers et al., 2018). New modes of framing and learning about forest stewardship complexities are required (González & Kröger, 2020).

Centering local knowledge and indigenous perspectives in land use research and planning decisions has the potential to catalyze the reductive and extractive problem‐solving of “science as usual” and “politics as usual” toward a more holistic, reciprocal and inclusive approach that understands humans to be a part of an interconnected whole—intrinsically linked to the health of the life systems that we both depend on and shape (Ban et al., 2018; González & Kröger, 2020; Ogar et al., 2020; Shepard Jr. et al., 2001).

### 1.2 The use of spaceborne lidar to measure ecosystem structure

Characterizing ecosystem structure with a co‐production approach offers a potential for addressing both the technical and the social challenges associated with monitoring and managing fragmented tropical landscapes. Forest structure can be defined as the horizontal and vertical distribution of vegetation surfaces in a forest including the trees, shrubs, and ground cover (including short vegetation and dead woody material) (Denslow & Guzman, 2000; DeWalt et al., 2003). Measures of forest structure allow for the direct estimation of aboveground biomass and carbon using allometric equations (Kellner et al., 2021). A growing body of work also documents how forest structure metrics are associated with various indicators of ecosystem functioning (Stark et al., 2015) and biodiversity (Aguilar‐Amuchastegui & Henebry, 2007; Bergen et al., 2009; Whitehurst, 2014; Wulder et al., 2012). Structural characterization of land cover types provides an entirely new source of information that can complement local information and other remote sensing approaches using optical imagery, which are limited by extensive cloud cover in the tropics.

Tropical forests are often considered the most structurally complex but least understood of forested ecosystems (Palace et al., 2016; Whitmore, 1982). Variations in tropical forest structure can be observed across continental (Malhi et al., 2014), landscape (Chazdon, 2017), and local scales (Norden et al., 2015). In addition to forest age (time since disturbance), a wide array of climatic and environmental factors also play a role in determining vertical and horizontal canopy structure (Arroyo‐Rodríguez et al., 2017; Brown & Lugo, 1990; Shaw, 2004). These factors include continental‐scale temperature and precipitation patterns; landscape‐scale influences of forest patch configuration, previous land use, disturbance regime, soil type, topography, and microclimate (Arroyo‐Rodríguez et al., 2017; Cook‐Patton et al., 2020; Crouzeilles et al., 2020; Shaw, 2004); as well as local‐scale processes of nutrient cycling, soil organic matter content, and stochastic successional community assembly dynamics (Feldpausch et al., 2007; Lebrija‐Trejos et al., 2010; Norden et al., 2015; Vandermeer et al., 2004). The dynamic variability in forest structure influences the extent to which tropical forests can provide the biodiversity, livelihood, and carbon sequestration that land stewardship planning efforts depend on.

Most forest structure studies to date focus on chronosequences and field experiments at the plot scale (Chazdon et al., 2007; DeWalt et al., 2003; Guariguata & Ostertag, 2001; Peña‐Claros, 2003) or airborne lidar (light detection and ranging) measurements at the local scale (Clark et al., 2021; Drake et al., 2002, 2003; Feldpausch et al., 2005; Palace et al., 2016; Stark et al., 2015). At both of these scales, many potentially important landscape factors remain relatively constant. In contrast, there is much less known about the structure of forest canopies across landscape, regional, and global scales (Clark et al., 2021; Feldpausch et al., 2005; Palace et al., 2016; Rödig et al., 2019).

With the advent of spaceborne lidar, it is now possible to sample forest structures at large scales (Dubayah et al., 2020; Wulder et al., 2012). Accurate three‐dimensional models of the forest can be constructed from spaceborne lidar laser pulses by analyzing the time delay from pulse emission to return (i.e., the time of interaction with forest targets of the ground, branches, and leaves). The returned energy of each waveform can then be processed to determine elevation, canopy height, and relative height metrics (Dubayah et al., 2020).

### 1.3 Co‐production of knowledge for tropical ecosystem stewardship

In addition to the drive for basic scientific understanding of tropical forest structure, we recognized an opportunity to design our project around the principles of “co‐production” (context‐based, pluralistic, shared‐goal oriented, and interactive) (Norström et al., 2020). In our project, this involved crafting research questions and carrying out the project objectives in a way that aligns with the priorities of all collaborators involved in our transdisciplinary team of local experts, community leaders, scientists, and land stewards (Baker et al., 2019; Cooley et al., 2022; Turnhout et al., 2020). We sought to move beyond “participation for legitimation” (top‐down consultation that simply informs a community of pre‐established land use research projects) as well as “participation for publication” (short‐term initiatives for research purposes only) (Cornwall, 2008; Rambaldi & Weiner, 2004). Instead, we focused on learning from and contributing to addressing the needs of local collaborators via iterative, dialog‐based interactions where all collaborators contributed resources and knowledge to the effort (Cornwall, 2008; Cornwall & Brock, 2005).

We engaged with local collaborators in Ucayali, Peru to learn about the aspirations and challenges of land stewardship and sustainability in the region. One of the priority needs articulated in these discussions was the ability to map common agricultural crops of the region—namely oil palm and cacao plantations—as well as various stages of forest degradation and regeneration that are frequently confused with high conservation value intact forest patches in land cover classifications that solely rely on passive remote sensing data. The ability to distinguish these land cover types provides a foundation for the enforcement of sustainable supply chain initiatives that numerous local and international organizations are in the process of creating in the region (Charry et al., 2020; Forest Carbon Partnership Facility (FCPF), 2019; GRDE, 2019a, 2019b; USAID, 2015).

### 1.4 Project objective and research questions

The overarching objective of our project was to assess the horizontal and vertical structural characteristics of the complex forest–agriculture interface in Ucayali, Peru. We defined three main research questions for our investigation to support our objective: (1) What vegetation classes describe the forest–agriculture dynamics in Ucayali, Peru? (2) What are the structural characteristics of these vegetation classes? (3) How can we employ a collaborative transdisciplinary approach that combines local knowledge and novel spaceborne lidar data to address questions (1) and (2)?

### 1.5 Hypotheses

**FIGURE 1:**
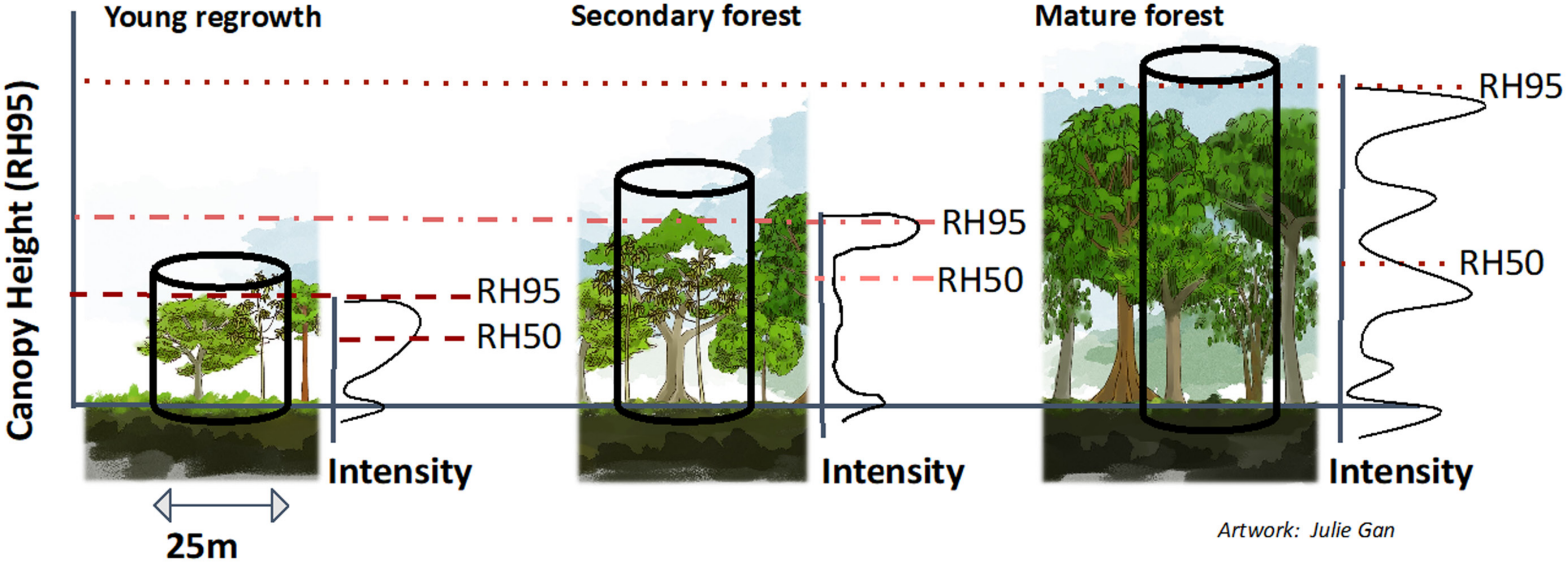
Hypothesized structural characteristics of each forest regrowth stage as captured by full waveform lidar observations. Forest regrowth stages span early succession (I), mid‐succession (II), and late succession or old‐growth (III). The hypothesized successional change in waveform‐derived relative height (RH) metrics of mean canopy height (RH95) and the height of median energy (HOME; RH50).

We hypothesized that young vegetation regrowth would have low canopy height, low variance in canopy height, a gradual increase in height across the cumulative energy profile, and a RH50:RH95 ratio higher than mature forest but lower than secondary forest (Figure 1). We hypothesized that mature lowland forests in Ucayali would exhibit the tallest canopy height and the most structurally consistent understory across all patches, with pervasive light limitation dynamics driving consistent canopy layering and gap formation reflected in low variation in cumulative energy curves. We predicted that secondary forests would have higher variation in canopy height due to differences in forest development stages across the forest patches we delineated. We also expected secondary forests to have RH50/RH95 and RH75/RH95 ratios closer to 1 compared to mature forests, which have lower canopy closure and a less dense understory.

Relative to mature and regenerating forests, we expected monocrop plantations to exhibit homogeneity in height and vertical canopy structure—assuming that tree spacing, soil conditions, and age are consistent in the plantation. Relative to the other agricultural classes (monocrop cacao and oil palm), we expected agroforestry cacao to exhibit the highest mean and standard deviation in canopy heights but the least structurally consistent signature across patches, as canopy layering depends on each farmer's choice of agroforestry tree species, stage of cultivation, spacing of trees, and pruning practices (Matocha et al., 2012). We anticipated lower structural variability in monocrop cocoa systems due to the absence of other tree species. However, we still expected monocrop cacao to exhibit substantially higher structural variability relative to oil palm due to the diverse set of pruning techniques and frequencies that our collaborating female farmers practiced.

## 2 METHODS

### 2.1 Case study: Ucayali, Peru

**FIGURE 2:**
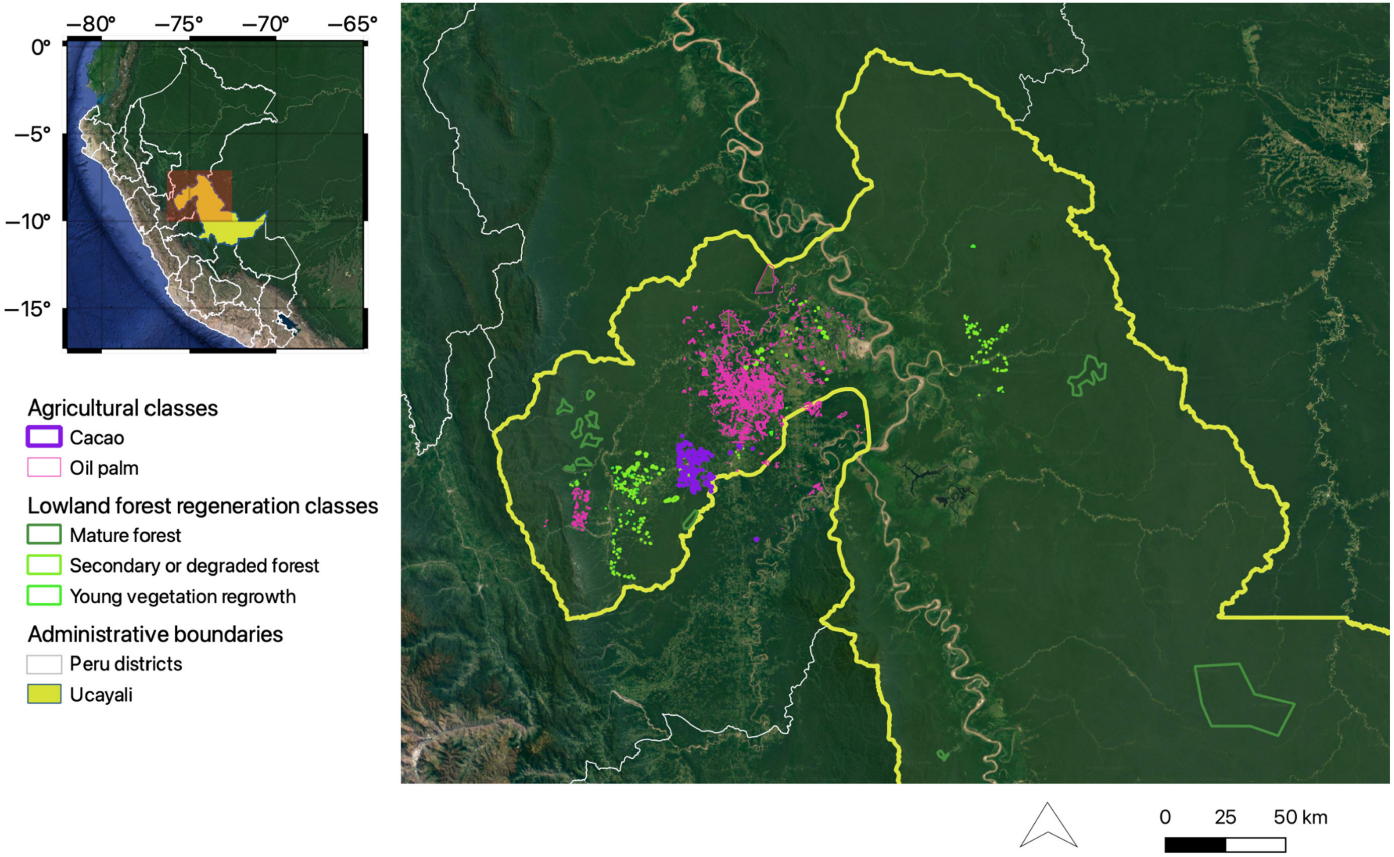
Study area of Ucayali, Peru, and delineated vegetation classes.

The case study we present here consisted of several land cover mapping activities carried out with a SERVIR‐Amazonia project in the Peruvian Amazon region of Ucayali (Figure 2). Ucayali has a tropical monsoon climate (Köppen classification: Am). We focused our work in northern Ucayali, which has an average annual rainfall of approximately 1560 mm (with an average of 114 rainy days) and an average annual daily temperature of 26.3°C (with mean daily minimum and maximum temperatures of 21.6°C and 31.1°C, respectively) (NOAA, 2022). The Ucayali region is named after the Ucayali River, a headwater tributary of the Amazon River. Approximately 2700 km long, the Ucayali River flows southward between the high Andes to the west and low hills to the east (Amazon Waters Alliance, 2022). Lowland rainforest occupies most of the Ucayali river basin up to approximately 1000 m, where montane forest begins to dominate (MINAM, 2018). Permanent and seasonally flooded forests grow along the Ucayali River. Our study did not include these inundated forest patches due to limitations in the ability of spaceborne lidar to detect “the ground” in continually changing inundation levels (Wulder et al., 2012).

Ucayali has a population of about 496,459 inhabitants (INEI, 2022). 87.6% of the population speak Spanish as a first language while 12.3% speak indigenous languages as a first language, including Asháninka (4.1%), Quechua (1.5%), Aymara (0.1%), and other indigenous languages, including Shipibo (6.6%) (INEI, 2007). Although we did not have direct connections to indigenous people in this first phase of the project, we began cultivating a relationship with an indigenous leader in the Shipibo‐Konibo tribe who we aspire to continue to learn from and collaborate with in future projects.

Ucayali is a unique area to study tropical forest regeneration dynamics because of the small‐scale, shifting agricultural cultivation patterns (Clavo Peralta et al., 2022; Dourojeanni, 1981; IRENA, 1996). These land use patterns sharply contrast with those in many other tropical areas with high rates of deforestation, which are often driven by large‐scale industrial agriculture (e.g., Brazilian Amazon and forests in Southeast Asia; Malhi et al., 2014). As a result of the small‐scale migratory agricultural land use, the landscape has a large variety of successional stages within a relatively small area compared to forests with forest regrowth from large‐scale industrial agriculture (MINAM, 2018). The crops of most economic importance in UcayaIi include rice, corn, cassava, beans, banana, papaya, coffee, cacao, and oil palm. Agricultural production of these crops combined increased 33% between 2014 and 2018, going from 654,992 tons in 2014 to 873,264 tons in 2018 (León Carrasco, 2019). Ucayali has four provinces: Atalaya, Purús, Coronel Portillo, and Padre Abad. We focused the data collection for this project in the latter two provinces due to existing collaborator ties to the region and because of the potential to directly support regional land stewardship planning policy in the most deforested provinces of Ucayali (MINAM, 2019).

### 2.2 Co‐production of land cover datasets

Local knowledge exchange complemented the remote sensing‐based approach to characterize the forest–agriculture regrowth dynamics in Ucayali. We co‐produced vegetation typologies with local collaborators who represented diverse groups including nonprofit organizations (Alianza Cacao, the International Center for Tropical Agriculture (CIAT), and Conservación Amazónica), local universities (Universidad Nacional de Ucayali and Universidad Nacional Mayor de San Marcos), regional and national government (Ucayali economic development office and the Servicio Nacional de Áreas Naturales Protegidas por el Estado, SERNANP) as well as women farmers cultivating cacao.

We also leveraged numerous reports and assessments published by national government (IRENA, 1996; MINAM, 2018, 2019), regional government (e.g., GRDE, 2019a, 2019b) as well as nonprofit organizations (Charry et al., 2020; Clavo Peralta et al., 2022; FCPF, 2019; Quiñones et al., 2018). Through a 2‐day workshop, subsequent informal dialogs occurring on a ~bi‐monthly basis for 2 years, and a targeted literature review, we learned about and documented how local experts, land stewards, and decision‐makers perceive and characterize the landscape.

**FIGURE 3:**
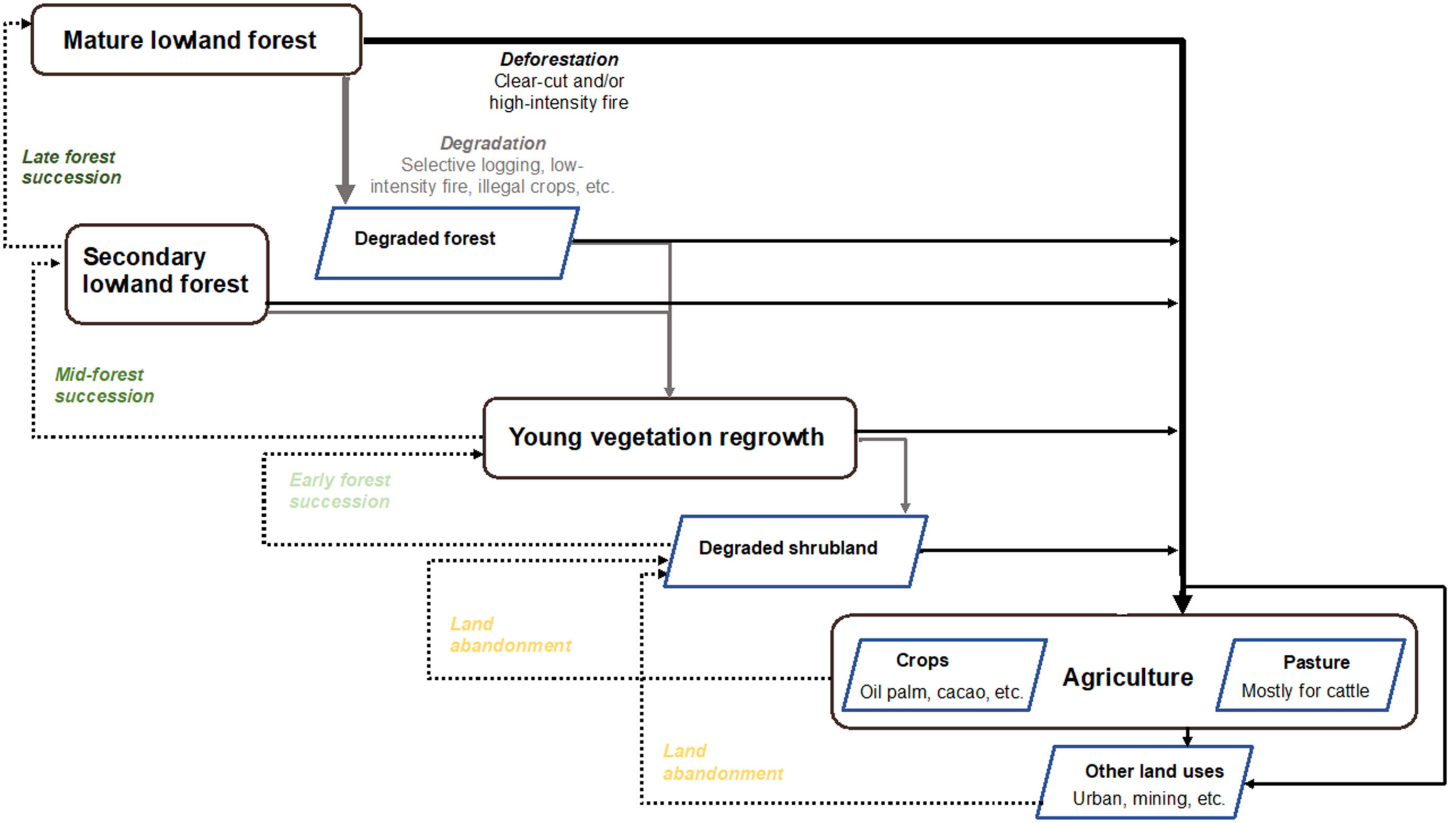
Flow diagram of vegetation regrowth dynamics in Ucayali with vegetation classes arranged in an order from least degraded (upper left quadrant) to most degraded (lower right quadrant). Dotted arrows represent instances of regrowth while unbroken arrows represent instances of deforestation (black) and degradation (gray). Bold arrows represent the most common pathways for mature lowland forest conversions, including both deforestation for agriculture and degradation for selective logging, low‐intensity burning, or illegal crop cultivation. The class names within the shaded green and yellow boxes represent the vegetation typologies identified for polygon delineation and subsequent waveform library development. These include: “Mature lowland forest,” “Secondary lowland forest,” “Young vegetation regrowth,” and “Agriculture” (oil palm, monocrop cacao, and agroforestry cacao are the focus of our subsequent analysis). The slanted blue parallelograms point to one or more land cover category(ies) that we do not focus on in this analysis but are nonetheless relevant for understanding the dynamic forest–agriculture interface of the region.

These inputs from our collaborators formed the basis of the iterative documentation of regrowth dynamics and the definition of vegetation classes used in our subsequent analysis (Figure 3). The lowland forest and vegetation regrowth typologies encompass the forest regeneration dynamics of small‐scale, shifting agricultural cultivation patterns that have characterized the lowland forests of the Ucayali region for over half a century (Dourojeanni, 1981).

### 2.3 Local vegetation classes in Ucayali

The development of the forest–agriculture vegetation class categories included written descriptions, georeferenced photos, and satellite imagery examples of each class (Table 1). The vegetation categories we focused on for subsequent polygon delineation included both forest and agricultural classes. We digitized polygons for each category of interest for the construction of class‐specific lidar waveform libraries. In particular, the categories included: (1) old‐growth lowland forest, (2) secondary lowland forest, (3) young lowland vegetation regrowth (“purma”), (4) mature oil palm plantations, and (5) cacao plantations (monocrop and agroforestry). The selection of these classes for digitization and subsequent waveform library generation arose from reconciling between technical feasibility (i.e., strengths and limitations of remote sensing capabilities) and local collaborator interest (i.e., what would be most useful for each of our collaborator organizations).

**TABLE 1:** Summary of the ecosystem and class names of the local vegetation typologies, including agricultural classes and lowland forest regeneration classes.

Nombre	Name	Description	Satellite imagery	Photograph captured from the ground
Cacao—monocultivo	Cacao—monocrop	Cacao (*Theobroma cacao*) monoculture involves the cultivation of only cacao trees in each field. It takes about 5 years (from seed) for a cacao tree to grow mature and start producing cacao pods	Description: Monoculture cacao plantation operated by smallholder women farmers in Ucayali. Source: MAXAR imagery 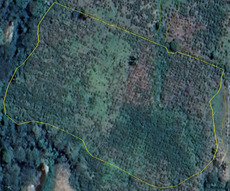	Description: Monoculture cacao plantation operated by smallholder women farmers in Ucayali. Source: Alianza Cacao/Juan Ayra Castillo (Facilitator) and Maria Teodocia Culque Leiva De Arquino (Farmer) 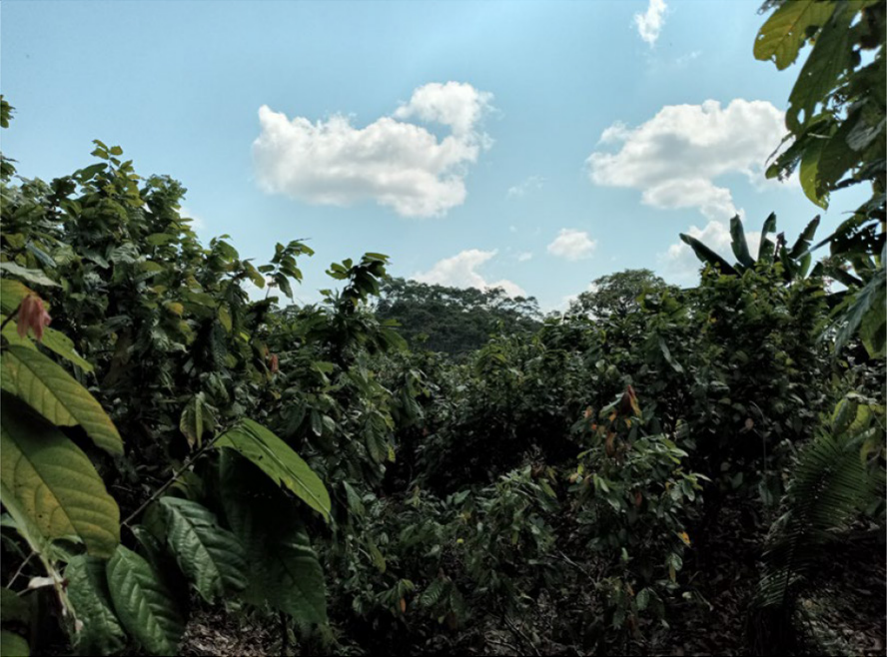
Cacao—agroforesteria	Cacao—agroforestry	Cacao agroforestry involves the cultivation of trees, shrubs, and other crops alongside cacao, as opposed to the production of one single crop. Trees commonly planted in cacao agroforestry systems include (1) leguminous tree species (*Gliricidia sepium*, *Erythrina poeppigiana,* or *Inga edulis*) used as mono‐specific shade in new cocoa plantations; (2) timber tree species (*Cordia alliodora*, *Terminalia ivorensis,* or *Tabebuia rosea*) used as mono‐specific shade in new cocoa plantations	Description: Agroforestry cacao plantation (Shaina Japanese Maple, sugar cane, and cacao) operated by smallholder farmers in Ucayali. Source: MAXAR imagery 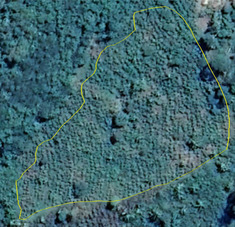	Description: Agroforestry cacao plantation (Shaina Japanese Maple, sugar cane, and cacao) operated by smallholder farmers in Ucayali. Source: Alianza Cacao/Florencio Capcha (Facilitator) and Irma Portillo Cruz (Farmer) 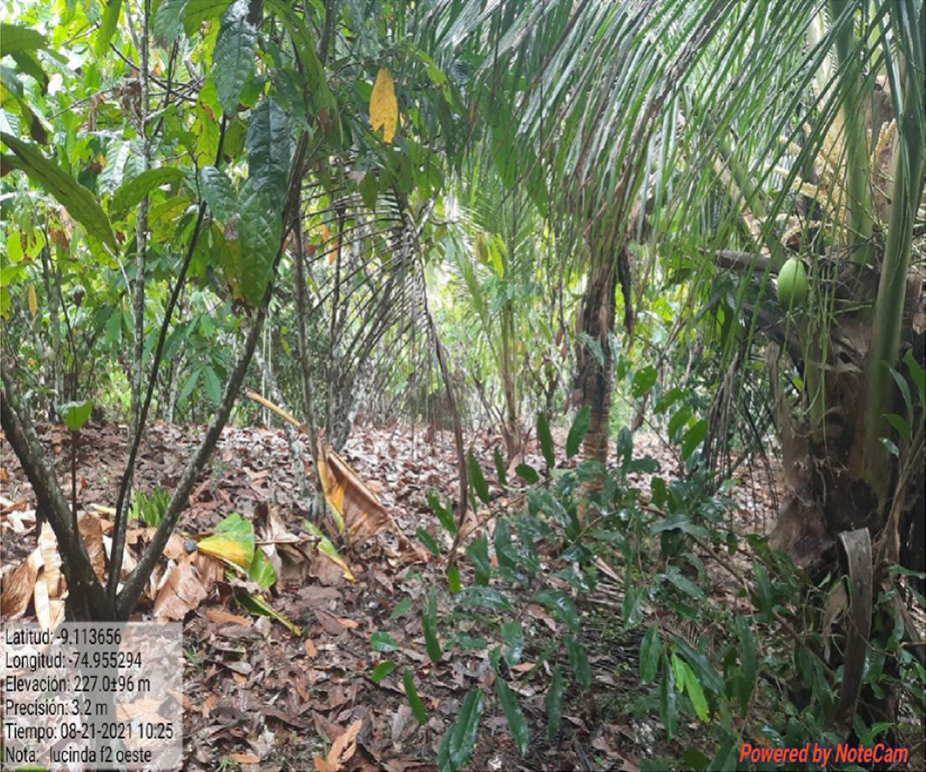
Palma aceitera	Oil palm	Oil palm (*Elaeis guineensis*) monoculture involves the cultivation of only oil palm trees in each field. It takes about 4 years (from seed) for an oil palm tree to grow mature and start producing fruit suitable for harvest	Description: Large‐scale industrial oil palm plantation. Source: Google Earth imagery 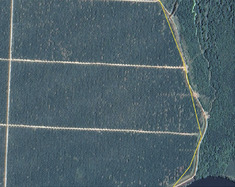	Description: Oil palm plantation operated by the Ocho Sur group in Ucayali. Source: Forest Peoples Programme/Matías Pérez Ojeda del Arco 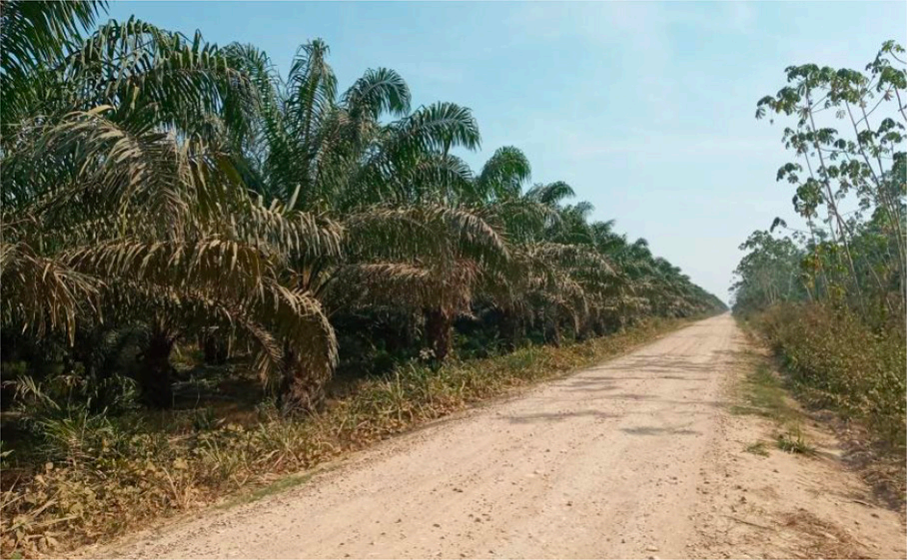
Bosque maduro de colina baja	Mature lowland forest	Old‐growth tropical forest (>20 years) in non‐inundated lowland regions	Description: Lowland forest in the buffer zone outside of Parque Nacional Cordillera Azul. Source: Bing Virtual Earth 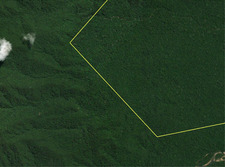	Description: Lowland forest in the district of Iparía. Source: SERNANP Ucayali department 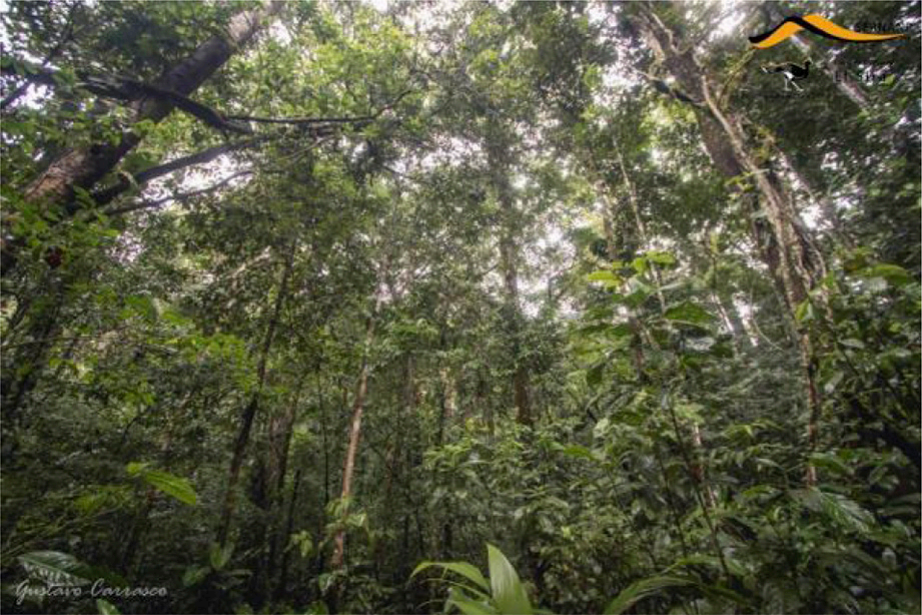
**“**Purma” o rebrote de vegetación joven	Young vegetation regrowth	Vegetation usually replacing recently abandoned agricultural land in non‐inundated lowland regions. Light green canopy color and mostly homogeneous canopy texture. Consists of herbaceous plants with some bushes and young trees, where pioneer species from the *Cecropia* genus and the *Trema* genus tend to dominate. No fully developed canopy cover but dense understory with age <~8 years since last abandonment	Description: Young vegetation regrowth. Source: Bing Virtual Earth 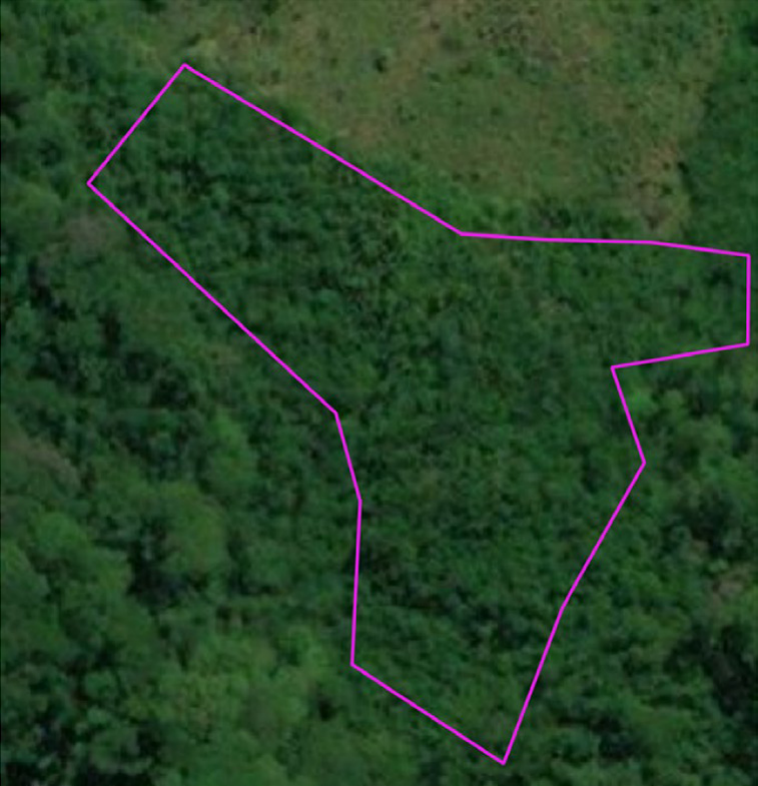 Description: Young vegetation regrowth. Source: Google Earth 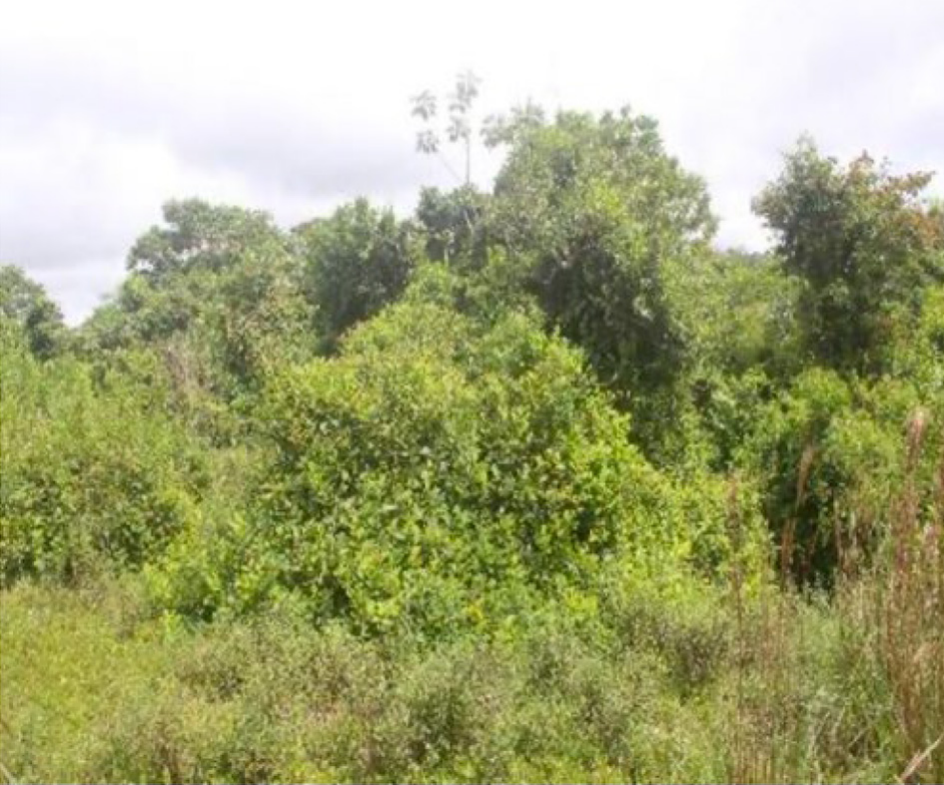	Description: Vegetation regrowth (5 years old). Photograph taken near San Alejandro. Source: Jorge Vela Alvarado, Universidad Nacional de Ucayali 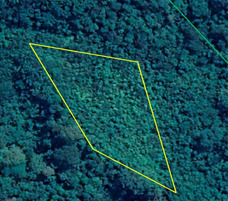 Description: Vegetation regrowth (3 years old) with some remnant plants from the previous cultivation period. Photograph taken in Huanuco, southwest of San Alejandro. Source: Alianza Cacao 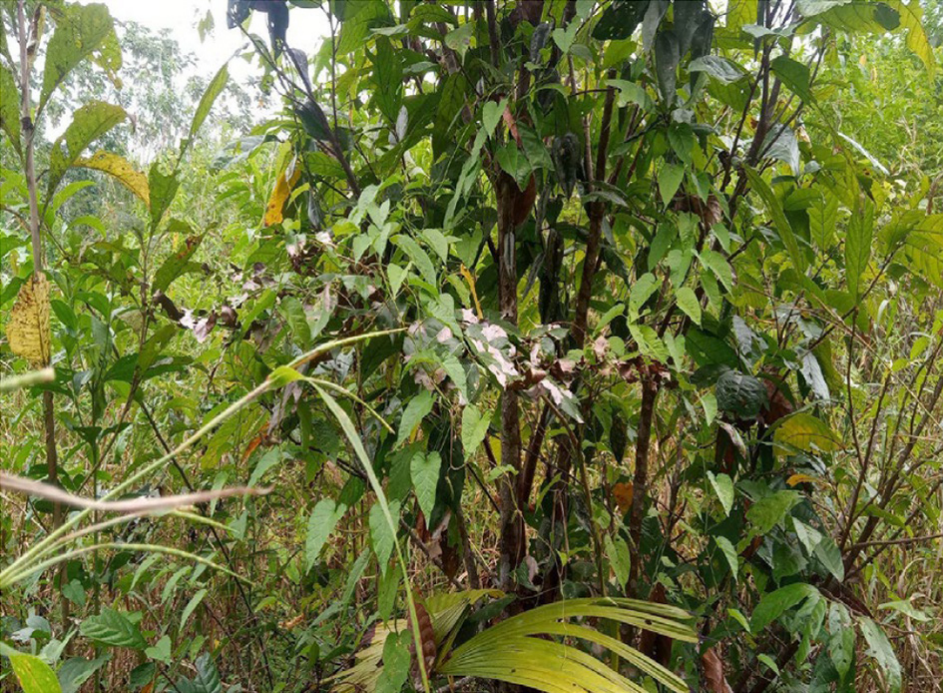
Bosque de colina baja secundario o degradado	Secondary lowland forest	Numerous layers of understory shrubs and mostly woody vegetation in non‐inundated lowland regions. Dense canopies with dark green color. Age ~8–20 years. Tree species of low commercial value, including many from the genus *Cecropia* and *Ochroma*	Description: Secondary forest (~10 years). Source: Google Earth 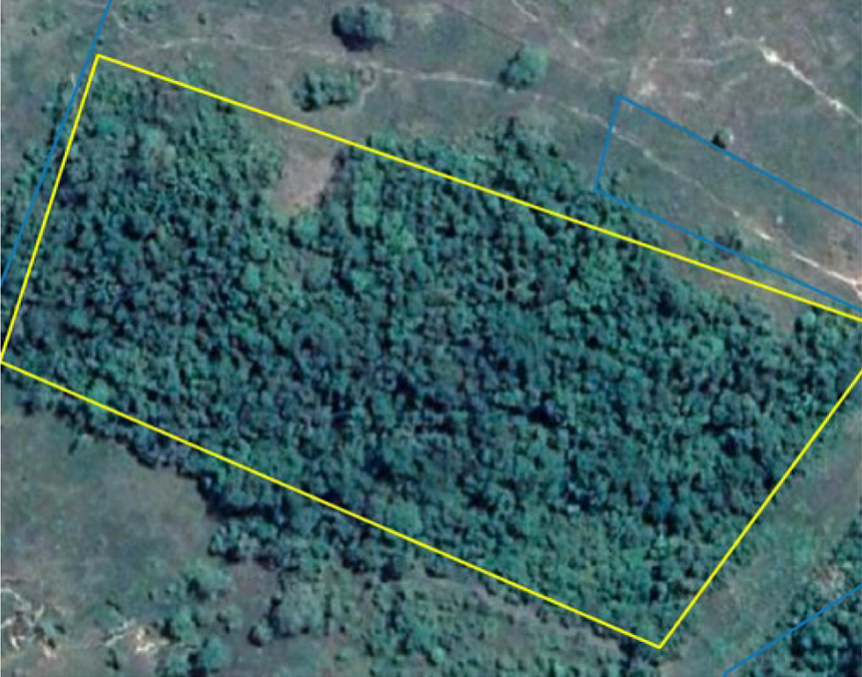 Description: Secondary forest (15 years). Source: Bing Virtual Earth 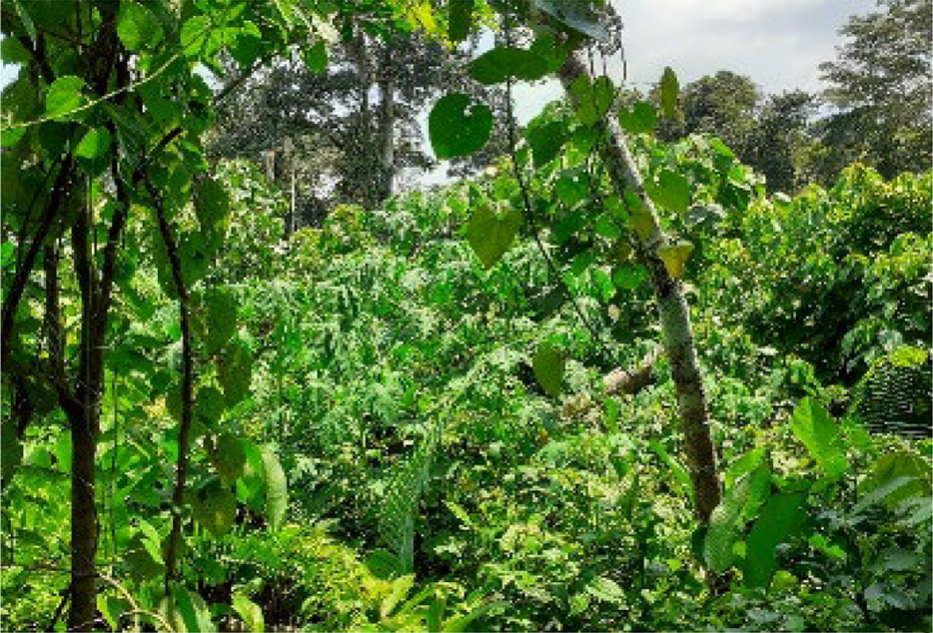	Description: Secondary forest (~10 years) with woody vegetation 5–7 m tall and dense understory shrubs. Photograph taken northwest of Mascho Piro Indigenous Reserve, Ucayali. Source: SERNANP 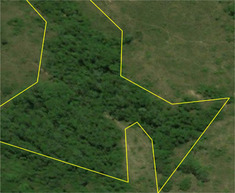 Description: Secondary forest (15 years) near San Alejandro, Ucayali, Peru. Source: Jorge Vela Alvarado, Universidad Nacional de Ucayali 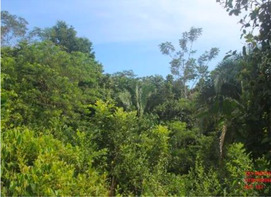

*Note*: Class names (Spanish and English), descriptions, examples from satellite imagery, and photographs taken from the ground corresponding to the local vegetation typologies. All locations are in latitude and longitude coordinates of the WGS 1984 (EPSG: 4326) geographic coordinate system.

#### 2.3.1 Forest regeneration

We focused on lowland forests because the lidar data to be used for waveform library signature generation are most accurate and reliable in nonmountainous but also nonflooded regions (Dubayah et al., 2020) and because these classes were most visually distinguished with the satellite base maps available. We delineated polygons for each of the classes via manual digitization based on local knowledge of the region coupled with visually perceived differences in greenness and canopy textures on the landscape with Google Earth, Bing, or MAXAR imagery. The spatial resolution of these imagery sources is anywhere between ~10 and 1.5 m resolution for Google Earth and Bing and ~0.3 m resolution for MAXAR. MAXAR imagery was only available for a subset of the geographic area of interest. Furthermore, Google Earth, and Bing imagery sources have low temporal resolution, with a range of years dating from ~2015 to 2019 associated with the mosaicked imagery tiles. For this reason, polygons that we digitized with Google Earth or Bing Virtual Earth required verification through time. We verified the land cover class consistency of the digitized polygons through time by visually inspecting each polygon with Planet imagery (monthly temporal resolution from 2016 to present). We down‐selected the original polygons, keeping only those that remained in their original class and removing all polygons that the Planet data indicated no longer had homogeneous dark green cover. The MAXAR imagery, on the other hand, was from 2020 and thus did not need temporal verification.

We also used two landsat‐based land cover classification time series datasets from (Reygadas et al., 2021). These data served as another check of our digitized polygons to ensure that all secondary and young vegetation regrowth polygons corresponded to areas that the landsat‐based algorithms also considered disturbed.

In parallel with polygon digitization, our collaborators contributed a number of georeferenced photos of each vegetation class captured from the ground. We engaged in regular, informal dialogs to continue to refine the vegetation class descriptions and polygons based on the input provided by local collaborators. For instance, our collaborators helped us gain confidence that our digitized secondary forest polygons did, in fact, correspond to secondary forest rather than degraded forest. In Ucayali, “degraded” forests typically correspond to selectively logged areas, which may experience a range of logging intensities and may still have some remnants of old‐growth species. Degradation may also occur due to fire (Uriarte et al., 2012). While we could not verify that such forms of degradation were totally absent in the polygons we digitized, we were confident that the large canopy gaps indicative of degradation were not present in the forest polygons we digitized. Furthermore, we learned that the high‐value wood targeted for selective logging is found in mature forests only. This provides further support for the separation of the secondary forest class as distinct from degraded forests.

#### 2.3.2 Oil palm

Delineation of oil palm polygons resembled the workflow described for the forest polygon dataset, except that we used Planet imagery for digitization and Bing and Google imagery for verification. We identified the large rectangular patches of plantations (regularly spaced trunks, consistent canopy of palm fronds symmetrically radiating out) with Planet imagery from June 2020, then verified with Bing imagery to confirm that the area was in fact oil palm rather than a different crop type.

Industrial plantations are typically led by commercial conglomerates and cover a large expanse of land. These plantations contain many fields that are connected by dirt roads and paths, while small holders are typically run by individuals or families and are much smaller fields in more circular or irregular shapes (Charry et al., 2020). When digitizing industrial plantations, we drew around the entire plantation, including every section and field that appeared to be connected to the same plantation. For small holders, we drew around the single fields we identified since there is uncertainty in how they connect or relate to surrounding small holders. To obtain a comparable, structurally consistent class, we focused the analysis on mature oil palm plantations only. After excluding all open canopy observations (i.e., young oil palm), we also excluded observations with canopy height lower than 8 m.

#### 2.3.3 Cacao

We mapped cacao plantations with the participation of women farmers who were either a producer, owner, or co‐owner of cocoa plantations in San Alejandro, Irazola district, Ucayali, Peru. This participatory mapping activity was developed with the support of Conservación Amazónica (ACCA) as a local partner in the Peruvian Amazon. All the participating women farmers were a part of the Alianza Cacao Peru (ACP) program. The Colpa de Loros Cooperative, which is a member of the ACP program, is located in the Monte Alegre‐Neshuya district, Padre Abad province. Huanuco and Ucayali regions were other important partner districts in the project's field activities.

First, the group of collaborators all coalesced around the shared objective to map cacao plantations. We then engaged in a dialog with all partners to solidify the aims and key concepts of the project, where we developed a proposed end‐to‐end approach to produce land cover maps. This presentation included an introduction to basic concepts of map making and remote sensing, allowing women farmers to understand how their knowledge fits in the larger process. Following this discussion, an interactive field sheet was prepared that allowed the collection of field data and provided visual material with photographs to further explain the fundamental land cover mapping concepts. The ACP technical team, who had previous experience collecting data in the field, shared information and images collected from the cocoa plantations at different stages with the facilitators. This allowed facilitators to provide the women farmers with clear examples of the information that the project aimed to collect during the project overview presentation.

The cacao plantations typically only formed a subset of the entirety of the property that the farmers owned. The women farmers shared their expertise about the different growth stages of cacao plantations and the ACP team provided images and field expertise. Coupled together, this information enabled the distinction between young and mature cacao plantations to be made when mapping the plantation locations within each farm.

### 2.4 GEDI data processing

The Global Ecosystem Dynamics Investigation is the first spaceborne lidar optimized for measuring ecosystem structure (Dubayah et al., 2020). Unlike the spaceborne lidar instruments from the Ice‐SAT missions, Global Ecosystem Dynamics Investigation (GEDI) has a nonpolar orbit on the International Space Station. This allows GEDI to collect many observations of forested areas in low and mid‐latitude regions of the earth. GEDI's unprecedented ability to measure forest structure is also because the wavelength of the instrument's laser pulses is in the near‐infrared, a portion of the electromagnetic spectrum where vegetation is particularly reflective.

Relative height (RH) metrics from GEDI define the percentage of the received laser waveform intensity that is less than a given height, where height is computed relative to the elevation of the lowest mode in the waveform. RH100 is the height of the highest return, which in theory is the maximum canopy height. In practice, it is more common to use RH95 as an estimate of canopy height due to issues distinguishing between signal and noise. RH50 is the height of median energy (HOME), that is, the height at which 50% of the energy of the waveform is returned. Metrics of HOME and all other RH percentiles, along with the ratios between them, depend on the ecosystem vertical structure. For example, if a forest has a thick canopy or a tall and dense understory, HOME and RH75 will be relatively closer to RH100 compared to a forest with a low understory and an upper canopy that does not have as much closure. As a result, the forest with a thicker canopy with more canopy closure (e.g., mature forest) would correspond to RH50/RH95 and RH75/RH95 ratios relatively closer to 1 compared to a forest with a low canopy closure (e.g., early secondary regrowth).

We obtained and processed data over our study region from the GEDI Level 2A Geolocated Elevation and Height Metrics product (GEDI02_A). We applied the following noise filtering steps based on the methodology outlined by Potapov et al. (2021): (a) power beam mode only, (b) beam sensitivity ≥0.9, and (c) ≤2 m range of predicted ground elevations among the five algorithms after removing the algorithm that yielded the largest outlier elevation. For (c), we deviated from Potapov et al. (2021) and chose a modified approach to retain some observations that otherwise would have been removed if applying the ≤2 m range threshold to all six algorithms. Our subsequent analysis focused on the subset of the high‐quality (i.e., noise‐filtered) GEDI data spatially overlapping the vegetation class polygon delineations.

We plotted cumulative energy curves and RH ratios across vegetation classes. To eliminate the influence of ground returns on subsequent analyses—particularly the RH ratio comparisons—we also computed above‐ground cumulative energy curves by removing observations with negative height values (i.e., removing the ground portion of the waveform). We linearly interpolated each observation from the remaining above‐ground observations to estimate the revised height metrics.

Our comparison between secondary and mature forest structures may have been influenced by the impact of canopy height on waveform signatures, where taller canopy height could allow for a relatively larger proportion of the energy from the waveform to correspond to the vertical forest profile versus the ground return. To test this, we computed a linear regression model consisting of RH75/RH95 as the dependent variable with vegetation class and canopy height (as well as confounding factors of elevation and spatial coordinates) as the independent variables (Appendix S1). Coordinates of points were included as interaction terms to account for spatial autocorrelation. Assumptions of homoscedasticity and linearity of fitted values versus residuals were generally met (Appendix S1 respectively). The assumption of normality of residuals was not met (Appendix S1 Shapiro‐Wilks *W* = 0.916, *p* < .0001). We therefore interpret these results with caution.

We recognized that edge effects in the data due to GEDI geolocation error may impact results. We observed that many of the GEDI observations that spatially coincide with the cacao plantations were not fully centered in the plantations but instead only partially overlapped with the plantations. We therefore removed edge effects using a larger ~65 m diameter homogeneous area (i.e., ±2σ geolocation error).

All data processing and statistical analyses were carried out using R version 4.1.2. (R Core Team, 2021) and all maps were made using QGIS version 3.22 (QGIS Development Team, 2022).

## 3 RESULTS

### 3.1 Cumulative energy curves among vegetation classes

**FIGURE 4:**
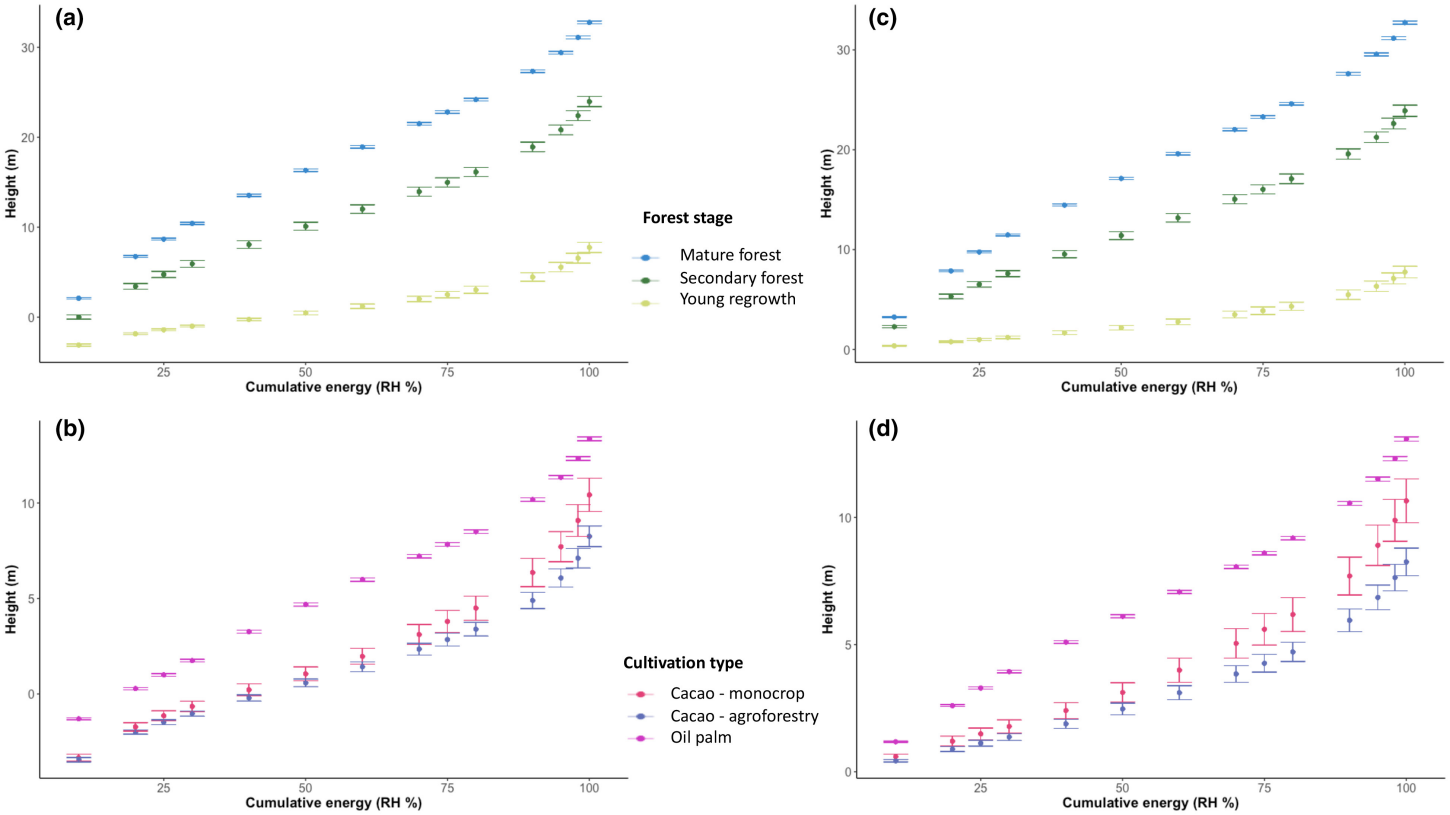
Mean and standard error of relative height (RH) metrics (i.e., cumulative energy levels) for (a, c) tropical lowland forest regeneration classes including mature forest, secondary, and young vegetation (“purma”); and (b, d) agricultural classes including oil palm plantations (all monocrop) and cacao plantations (both monocrop and agroforestry). Negative height values represent the ground returns in each observation, which arises due to low canopy cover. Panel (b) shows the results from removing the negative height returns and interpolating each observation from the remaining above‐ground observations.

Plotting the cumulative energy of each vegetation class reveals a basic pattern present across all classes: heights increasing more gradually at low RH percentiles and more steeply toward higher RH percentiles (Figure 4a,b and Appendix S1). This pattern is particularly pronounced in vegetation classes with low canopy height (i.e., cacao plantations and young vegetation regrowth). Secondary lowland forests exhibit a similar vertical profile to mature lowland forests across all RH percentiles. Young vegetation regrowth forest class exhibits a much more gradual increase in height across low RH percentile, where the largest changes in heights occur only at RH90–RH100. The cumulative energy plots for agroforestry and monocrop cacao plantations also closely resemble one another: gradual increases at low RH values followed by a steep incline at high RH percentiles. Meanwhile, oil palm RH heights increase more consistently across all percentiles.

**FIGURE 5:**
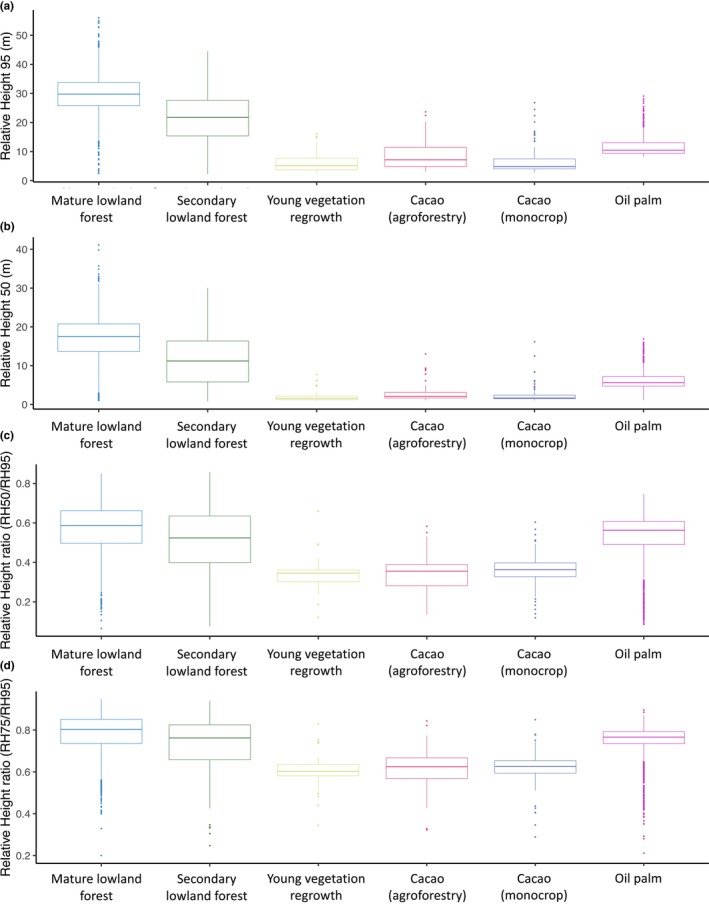
Relative height (RH) metrics and ratios across vegetation class types: (a) mean canopy height (RH95), (b) the height of median energy (HOME; RH50), (c) the ratio of HOME to canopy height (RH95) (RH50/RH95), and (d) the ratio of RH75 to canopy height (RH95) (RH75/RH95). All data reported in this graph have been transformed to eliminate the influence of ground returns on the results.

We found pronounced differences in the proportion of ground energy in the waveforms of cacao and young vegetation regrowth observations (above‐ground energy returns start after ~RH35) versus oil palm, mature forest, and secondary forest classes (above‐ground energy returns start after ~RH10) (compare Figure 5 with Appendix S1). The ground return removal analysis did not change the major patterns of the cumulative energy curves (Figure 4c,d).

**FIGURE 6:**
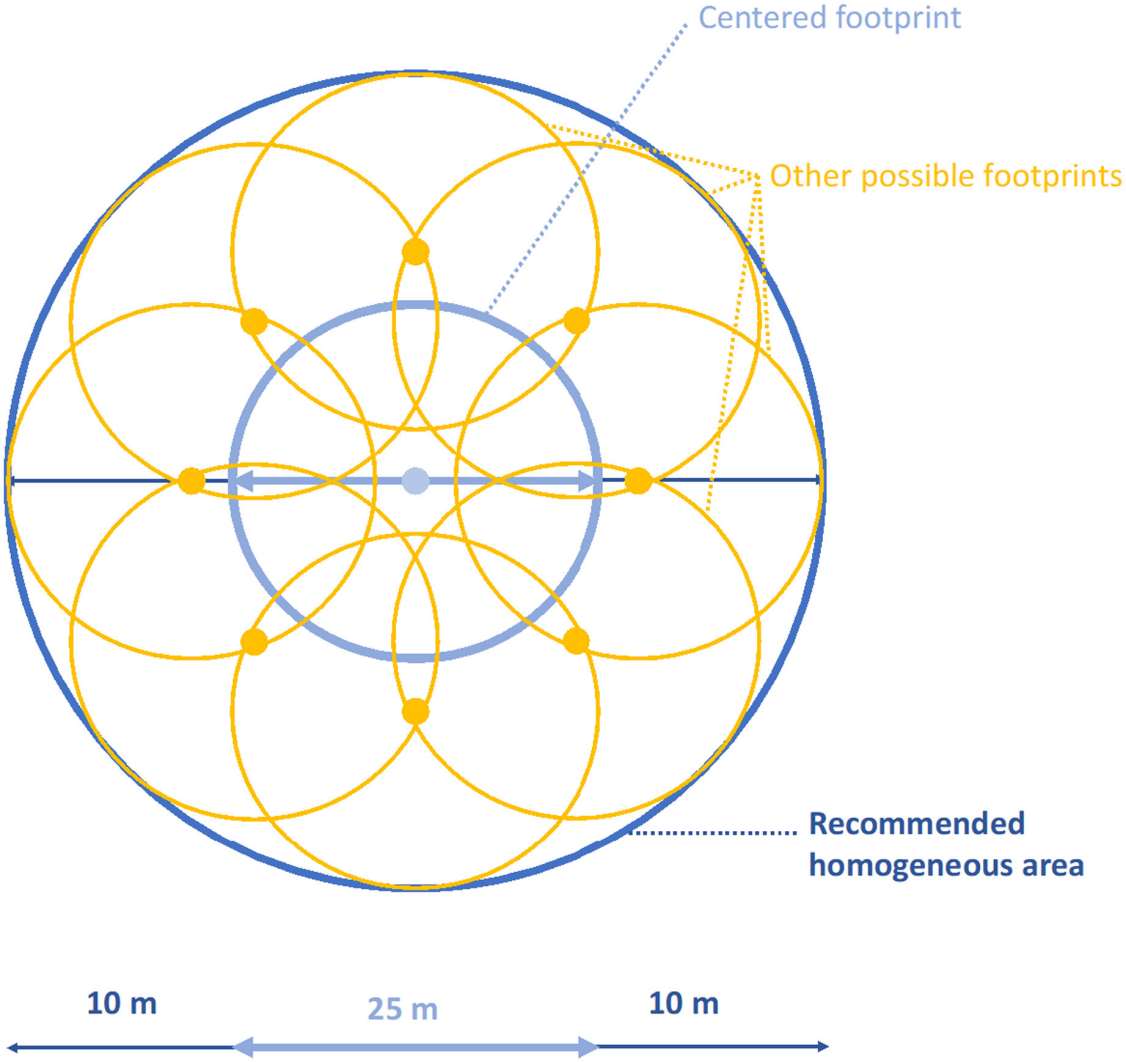
Illustration of the need to expand the range of GEDI observations to include a footprint with a diameter of at least 45 m (25 m of the original diameter area of the beam, and ±10 m additional buffer to account for geolocation error). In applications that aim to characterize distinct forest types and/or successional stages of regeneration, it is especially important to consider a 45 m diameter area for each GEDI observation, where special attention must go toward ensuring homogeneity of the forest patch within the 45 m diameter footprint (i.e., eliminating observations where multiple forest types or edges occur in the larger conservative footprint area).

**FIGURE 7:**
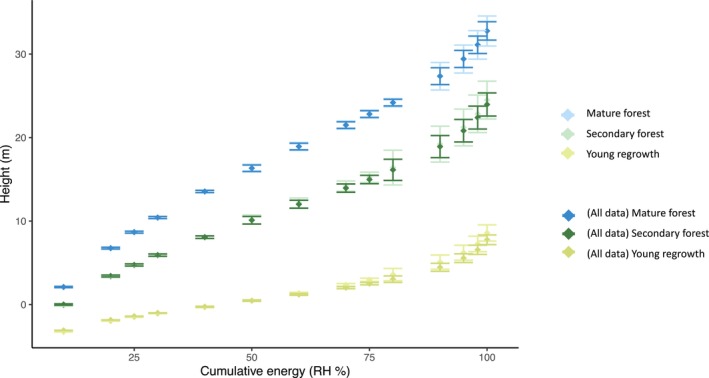
Characteristic waveforms (mean and standard error of relative height metrics) when edge effects are removed using a conservative ±20 m or 2σ error (light colors) versus when all data are included (darker colors) for tropical lowland forest regeneration classes (mature forest, secondary forest, and young vegetation).

Furthermore, we removed edge effects in our dataset using a larger ~65 m diameter homogeneous area (i.e., ±2σ geolocation error; Figure 6). After this filtering, no observations remained in the cacao dataset and fewer observations remained in the forest stage dataset. Nonetheless, the overall structural patterns shown in the relative heights of each forest stage remained very similar (Figure 7).

### 3.2 Canopy height (RH95) and height of median energy (RH50)

Overall, mature lowland forests exhibited the tallest mean canopy height as quantified by RH95 (29.55 m) relative to both of the other forest classes (secondary lowland forest is 21.25 m and young vegetation regrowth is 6.33 m) as well as the agricultural classes (cacao – monocrop is 6.85 m, cacao – agroforestry is 8.9 m and oil palm is 11.5 m; Table 2a). The highest standard deviation in canopy height arose in the secondary forest class (8.85 m) while the lowest standard deviation in canopy height occurred in the monocrop mature oil palm plantations (3.07 m) closely followed by the young vegetation regrowth class (3.56 m). These results generally remained consistent with the values observed before transforming the data to eliminate the influence of ground returns on the results (Table 2b).

**TABLE 2:** (a) Summary statistics of canopy height (RH95 in m) for each vegetation class in this analysis: Agricultural classes including oil palm plantations (all monocrop) and cacao plantations (both monocrop and agroforestry); and tropical lowland forest regeneration classes including mature forest, secondary and young vegetation. (b) Summary statistics of RH95 from the original, untransformed data (i.e., the ground returns are still influencing the results).

(a)								
Land cover	Max	Min	Q25	Median	Q75	Mean	SD	Count
Cacao – agroforestry	23.66	3.03	4.86	7.15	11.44	8.9	5.36	46
Cacao – monocrop	26.78	2.85	4.03	4.83	7.45	6.85	4.81	97
Mature lowland forest	55.98	2.5	25.77	29.76	33.8	29.55	6.78	2063
Oil palm	29.08	8.22	9.37	10.47	13.04	11.5	3.07	1503
Secondary or degraded lowland forest	44.48	2.22	15.37	21.74	27.62	21.25	8.85	282
Young vegetation regrowth	16.13	2.58	3.73	5.15	7.71	6.33	3.56	46

(b)								
Vegetation class	Max	Min	Q25	Median	Q75	Mean	SD	Count
Cacao – agroforestry	23.48	0	4.23	5.68	9.63	7.71	5.4	47
Cacao – monocrop	26.61	2.32	3.4	4.15	6.5	6.07	4.74	97
Oil palm	33.97	8.01	9.09	10.3	12.48	11.28	3.28	1612
Mature lowland forest	55.98	2.05	25.51	29.65	33.71	29.40	6.89	2061
Secondary lowland forest	44.48	1.87	14.94	21.35	27.3	20.82	9.15	281
Young vegetation regrowth	15.65	2.13	3.07	4.22	6.64	5.55	3.55	46

*Note*: The data represented have the negative height returns removed (i.e., the ground returns are not influencing the results).

Similar to canopy height, mean RH50 values were highest for mature lowland forests (17.13 m), followed by secondary lowland forests (11.41 m) (Figure 5 and Table 3a). The differences between results from the transformed data (Table 3a) versus the original data (Table 3b) are much greater for RH50 than for RH95. Young vegetation regrowth closely resembled the monocrop cacao and agroforestry cacao classes, with mean RH50 values of ~2–3 m (2.19, 2.47, and 3.12 m respectively).

**TABLE 3:** (a) Summary statistics of the height of median energy (RH50 in m) for each vegetation class in this analysis: Agricultural classes including oil palm plantations (all monocrop) and cacao plantations (both monocrop and agroforestry); and tropical lowland forest regeneration classes including mature forest, secondary and young vegetation. (b) Summary statistics of RH50 from the original, untransformed data (i.e., the ground returns are still influencing the results).

(a)								
Land cover	Max	Min	Q25	Median	Q75	Mean	SD	Count
Cacao – agroforestry	13.04	1.09	1.66	2.06	3.08	3.12	2.58	46
Cacao – monocrop	16.17	1.02	1.45	1.66	2.4	2.47	2.24	97
Mature lowland forest	41.11	1.03	13.67	17.47	20.78	17.13	5.52	2063
Oil palm	16.88	1.14	4.72	5.6	7.17	6.11	2.35	1503
Secondary or degraded lowland forest	29.98	0.82	5.8	11.2	16.34	11.41	6.67	282
Young vegetation regrowth	7.75	0.89	1.25	1.59	2.17	2.19	1.6	46

(b)								
Land cover	Max	Min	Q25	Median	Q75	Mean	SD	Count
Cacao – agroforestry	11.94	−1.19	−0.03	0.26	0.82	1.06	2.47	47
Cacao – monocrop	14.05	−1.45	−0.18	0.03	0.52	0.58	1.96	97
Mature lowland forest	41.11	−1.38	12.85	17.11	20.64	16.33	6.35	2061
Oil palm	20.29	−0.37	3.03	4.64	6.32	4.93	2.85	1612
Secondary or degraded lowland forest	29.98	−2.02	2.58	10.6	15.65	10.1	7.57	281
Young vegetation regrowth	6.02	−0.85	−0.21	−0.01	0.36	0.46	1.33	46

*Note*: The data represented have the negative height returns removed (i.e., the ground returns are not influencing the results).

Mature and secondary forests showed higher standard deviation in RH50 values—almost as large in magnitude as the standard deviation in canopy height. In secondary lowland forests, for instance, we observe mean RH50 to be about ½ mean RH95 (11.41 m/21.25 m = 0.54). Yet the standard deviation of RH50 is nearly the magnitude of the standard deviation of RH95 (compare sd RH50 = 6.67 m with sd RH95 = 8.85 m), resulting in a ratio of ~0.75 (6.67 m/8.85 m). The ratio of 0.75 is greater than the ratio of around 0.5 that would arise if scaling of standard deviation across height were consistent. This indicates that secondary lowland forests exhibited more heterogeneity in terms of their vertical structure as the heterogeneity they exhibit in total canopy height. We found similar magnitudes in sd ratios for oil palm and mature forest.

We suspected that lower RH50/RH95 ratios in the secondary forest class could possibly be attributed to the artifact of the lower mean canopy height in this class driving a relatively larger portion of the waveform energy to correspond to the ground signal. We found a positive and statistically significant effect of canopy height on RH50/RH95 (RH95 coefficient = 0.0073, *p* < .0001). After accounting for the effect of height, mature forests still have higher RH50/RH95 ratios compared to secondary forests we measured (mature forests RH50/RH95 was ~0.03 higher relative to secondary forests, *p* < .0001). Thus, our original hypothesis of a higher RH50/RH95 ratio for secondary forests is not supported by our data, even when accounting for the influence of canopy height on the RH ratios.

## 4 DISCUSSION

### 4.1 Ecosystem structure in the forest–agriculture interface

Here, we plotted relative height profiles for forest and agricultural regions in Ucayali, Peru. We found that secondary lowland forests exhibit a similar vertical profile to mature lowland forests, with each relative height percentile increasing at similar rates throughout the cumulative energy profile (Figure 4). This similarity in cumulative energy profiles was surprising. Correspondingly, results indicate that even after accounting for the effect of canopy height on relative height metric ratios, the mean ratio of HOME to canopy height (RH50/RH95) are highest secondary lowland forest (0.53) closely followed by mature lowland forests (0.54) (Figure 5c,d and Table 4). Even if degradation were not fully separated and removed from the secondary forest class, we would still expect the vertical structure to have a distinct distribution of biomass along the vertical column relative to mature forest due to well‐documented structural differences in tropical forest successional stages (Figure 1; Budowski, 1970; Denslow & Guzman, 2000; Guariguata & Ostertag, 2001; McElhinny et al., 2005). However, that is not what we found.

**TABLE 4:** (a) Comparison of the ratios of mean RH50 (m) to mean RH95 (m) versus sd RH50 (m) to sd RH95 (m) for each vegetation class in this analysis: Agricultural classes including oil palm plantations (all monocrop) and cacao plantations (both monocrop and agroforestry); and tropical lowland forest regeneration classes including mature forest, secondary, and young vegetation. (b) Summary statistics of RH50 from the original, untransformed data (i.e., the ground returns are still influencing the results).

(a)							
Vegetation class	Mean RH95	SD RH95	Mean RH50	SD RH50	Ratio Mean RH50/95	Ratio SD RH50/95	SD RH50/95 – Mean RH50/95
Cacao—agroforestry	8.9	5.36	3.12	2.58	0.35	0.48	0.13
Cacao—monocrop	6.85	4.81	2.47	2.24	0.36	0.47	0.11
Mature lowland forest	29.55	6.78	17.13	5.52	0.58	0.81	0.23
Oil palm	11.5	3.07	6.11	2.35	0.53	0.77	0.23
Secondary lowland forest	21.25	8.85	11.41	6.67	0.54	0.75	0.22
Young vegetation regrowth	6.33	3.56	2.19	1.6	0.35	0.45	0.10

(b)							
Vegetation class	Mean RH95	SD RH95	Mean RH50	SD RH50	Ratio Mean RH50/95	Ratio SD RH50/95	Diff. mean and sd ratios
Cacao—agroforestry	7.71	5.4	1.06	2.47	0.14	0.46	0.32
Cacao—monocrop	6.07	4.74	0.58	1.96	0.10	0.41	0.32
Mature lowland forest	11.28	3.28	4.93	2.85	0.44	0.87	0.43
Oil palm	29.4	6.89	16.33	6.35	0.56	0.92	0.37
Secondary lowland forest	20.82	9.15	10.1	7.57	0.49	0.83	0.34
Young vegetation regrowth	5.55	3.55	0.46	1.33	0.08	0.37	0.29

Typically, early successional regrowth exhibits low, homogeneous vegetation height and high stem density, with the dominance of young pioneer species that have minimal woody biomass and no developed canopy (Budowski, 1970; Denslow & Guzman, 2000; Guariguata & Ostertag, 2001). Structurally, we expected this stage to have low canopy height with low variance of height across space (i.e., high textural homogeneity) and no distinguishable canopy layers (Clark, 1996; Drake et al., 2003; McElhinny et al., 2005). This is, in fact, generally what we observed: low variance in canopy height, a gradual increase in height across the cumulative energy profile, and an RH50:RH95 ratio lower than secondary forest. Meanwhile, the mean and variability in the young vegetation regrowth and cacao classes were similar, with the magnitudes of the interquartile ranges of RH50/RH95 between ~0.3 and 0.4.

In contrast, mid‐successional regrowth generally exhibits a taller, more established canopy, clearer understory due to light competition and stem exclusion as well as emerging distinct canopy layers (Denslow & Guzman, 2000; Drake et al., 2003; McElhinny et al., 2005). Late‐successional forest (i.e., mature forest) has a fully closed canopy and typically features distinct canopy layering. Though secondary forests in advanced stages of stem exclusion eventually exhibit canopy layers and other structural similarities to mature forest (e.g., see Poorter et al., 2021), we still expected the secondary forest class to exhibit a higher RH50:RH95 ratio and a more steeply increasing cumulative energy curve relative to mature forest due to differences in canopy closure and lower tree species diversity (Drake et al., 2002; MINAM, 2018, 2019; Whitehurst, 2014).

Among forest classes, mature lowland forest was consistently taller but had lower standard deviation than secondary lowland forest (Table 2 and Figure 5). The lower variance in canopy height of mature forests followed our expectations. This could be attributed to the fact that the secondary forest patches correspond to a range of forest development ages and stages. The structural heterogeneity likely also reflects the small‐scale, high‐frequency disturbance regimes causing these areas to become a complex mosaic of regeneration stages. We did not focus on forest age as a main distinguishing factor among the three regenerating forest classes because major structural discrepancies can exist in forests of the same age even if they are neighboring patches due to local environmental heterogeneity as well as complex community‐ and species‐level interactions (Chazdon, 2003; Norden et al., 2015; Rozendaal & Chazdon, 2015). Rather, we isolated forest *stages* of regrowth by identifying forest patches that fit a locally defined description of visually observable canopy texture and spectral characteristics. This approach still yielded forest patches that vary substantially in height from one patch to the other, even if the heterogeneity of canopy height within each secondary patch is lower than the heterogeneity (i.e., variation) of canopy height within a mature forest patch of equal area and approximately comparable environmental conditions.

We found that the standard deviations of RH50 were nearly equal in magnitude to the standard deviations of RH95 for secondary forest (sd sRH50 of 6.67 and sd of RH95 of 8.85), mature forest (sd of RH50 of 5.52 with sd of RH95 of 6.78) and oil palm (sd RH50 of 2.35 and sd of RH95 of 3.07). For oil palm, we interpret high standard deviation ratios to arise due to variation in the canopy closure of the palm trees, where plantations with high canopy closure result in RH50 values close to canopy height while plantations with lower canopy closure result in RH50 values close to the ground (due to a lack of sufficiently large shrubs and trees to form an understory). The high standard deviation of RH50 relative to the standard deviation of RH95 that we observed in secondary forests further underscores the presence of numerous stages of regrowth in this vegetation class along with the possible presence of degradation in some secondary forest patches as mentioned above. Yet we also found that mature forests exhibit high variation in their vertical structure. We were surprised that this variation was approximately the same magnitude as secondary forest. To the best of our knowledge, the mature forest patches selected for this study did not recently experience any major disturbance event. Thus, the similarity in vertical structure to secondary forests indicates variation in the understory structure. Relatively higher standard deviation in RH50 could arise because of high variation in structural organization of the understory. Unless a rare, naturally occurring canopy gap is present due to the mortality of a large tree (Farrior et al., 2016), we expected the mature forest patches in this analysis to share a high structural resemblance in the understory, with similar number and spacing of canopy layers due to comparable levels of light limitation (Denslow & Guzman, 2000). However, our results suggest that the distribution of biomass in the understory of the mature forests we studied has high variation, nearly as high as the variation in canopy height. This may suggest that variables other than light limitation—such as nutrient availability and microtopography—may be playing an important role in driving the structure of the understory in mature lowland forests. Such an investigation is an important area for future work.

It is possible that the secondary forest class did not fully differentiate between “degraded” and “secondary forest” due to limitations in the digitization workflow, which relied on visual interpretation of satellite imagery and cross‐comparison with Landsat‐derived forest regeneration time series data (Reygadas et al., 2021). Low‐intensity disturbances that influence the understory of the canopy (e.g., low severity fires) could be present in the secondary forest patches. However, the cumulative energy plots (Figure 4) could provide some further support for our initial assertion that only minimal forest degradation was present, if any at all. If high severity degradation were present in the secondary forest patches, then the canopy closure would be patchy and the vertical profile would show some ground returns similar to the agricultural classes of cacao (i.e., low RH percentiles corresponding to heights <0 m). We would also expect this to translate into vertical profiles with high variation, which would be easier to detect in mature forests but still likely detectable in secondary forests. The high variation in vertical cumulative energy profiles would arise due to a split in structural characteristics in degraded versus nondegraded secondary forest patches (even if our relative sample size of secondary/degraded forest class increased to be close to the much larger sample size of the mature forest class). In particular, we would expect rapid increases in cumulative energy of the waveform for forest patches with high canopy closure and much slower increases for degraded patches that do not have full canopy closure. Instead, the lowest portions of the cumulative energy curve (i.e., low RH values) in the secondary forest class all corresponded to heights above the ground and the standard error bars remained low across all cumulative energy levels.

### 4.2 Co‐production and participatory land cover mapping

While our co‐production approach was based on the findings of land cover mapping initiatives in the Amazon as well as existing citizen science‐based land cover mapping literature, our lessons learned could apply to many other land cover mapping projects globally. These include: (1) Create a shared vision, outcomes, and expectations for the collaborative project; (2) Co‐produce joint concepts and land cover keys; and (3) Cultivate a culture of two‐way knowledge exchange. Please refer to Supplemental Information Section 2 for an explanation and set of examples of each of these.

The results from our project contribute toward the enforcement of the high‐priority monitoring areas established by the Ucayali regional government land stewardship plan (Gobierno Regional de Ucayali, 2022). Titled “Ucayali Regional Strategy for Low Emissions Rural Development,” the plan expresses the desire of the regional government to reduce deforestation rates by improving the value‐chain of key crops. The plan identified priority monitoring areas based on a baseline binary forest versus nonforest map combined with a spatial analysis of factors associated with deforestation. The results suggested that these at‐risk areas occupied 11.35% of Ucayali's intact forests (1,059,312 ha). Gaining nuanced structural distinctions among forest regeneration stages allows for prioritization of which at‐risk forest patches to most closely monitor and protect.

Many of these at‐risk areas coincide with potential agricultural expansion areas. The Ucayali regional government Office for Economic Development, for instance, established zoning boundaries for potential oil palm plantations (Charry et al., 2020; GRDE, 2019a) as well as cacao plantations (GRDE, 2019b). The zoning aims to constrain the spatial expansion of potential new oil palm and cacao plantations to areas that have already been deforested and/or heavily degraded. However, these areas may still have some remaining high conservation value patches of old‐growth and mature secondary forest. The Ucayali Office for Economic Development expressed interest in developing streamlined approaches for monitoring these areas to ensure that new cacao and oil palm plantations (1) do not occur outside of the zoning boundaries and (2) do not replace any existing high conservation value secondary forest fragments within the boundaries. Our research can support these two goals by integrating forest structure information into enforcement procedures of comparing current land cover conditions to baseline zoning maps. Specifically, lidar‐based forest structure data allow the Ucayali regional government to overcome regional‐scale detection challenges of differentiating high conservation value secondary forest versus lower conservation value young vegetation regrowth, which are easily confused in land cover classification models that solely rely on optical data (Gobierno Regional de Ucayali, 2022). Thus, our project contributes toward the enforcement of high‐priority monitoring areas in the agriculture–forest interface established by the Ucayali Government.

### 4.3 Limitations and future work

We showed that the GEDI geolocation error affects our ability to accurately characterize small‐scale cacao plantations. In addition to cacao mapping, this limitation of GEDI impacts the application to Ucayali's zoning enforcement by limiting the utility of monitoring small patches of high conservation value forest. This limitation of GEDI data further underscores the opportunity of *combining* remote sensing data with local knowledge to identify high conservation value secondary forest patches, particularly those smaller than a ~65 m diameter area (i.e., ±2σ geolocation error).

One other limitation we note is the nonrandom spatial distribution of GEDI data. The nonrandom distribution of observations arises due to the nature of GEDI's orbit, data collection tracks as well as noise removal processing. Many tropical regions have particularly sparse coverage, including some areas of the western Amazon (Potapov et al., 2021). As a result, it is possible that our dataset misses sparse large trees in high‐conservation value secondary forest patches.

Lidar also has limitations in forests with dense canopies, where the laser cannot penetrate the canopy as fully as ecosystems with more open canopies. This effect can cause waveform profiles to not reach the ground and/or waveform profiles that do have a ground return but whose cumulative energy curves have steep initial slopes at small RH values. In the latter case, this would be reflected by RH 50 values close to RH95 values (i.e., RH50/RH95 ratios close to 1). We found that the mean RH50/RH95 ratio in mature forests and oil palm—the vegetation classes with the densest canopies—were both much lower than 1 (0.53 and 0.58, respectively). This provides evidence that the saturation effect of dense canopies was generally not a concern in our results. It further suggests that the noise filtering we did (see 2. Methods) was effective, including the beam sensitivity threshold of ≥0.9.

We acknowledge that collaborator participation in the project was not driven from the single motivation of generating the land cover data. Rather, participants collaborated for a range of reasons. For example, many of the women farmers who participated in the cacao plantation mapping activity named motivations including altruism, self‐achievement, curiosity, personal interest in technology, and self‐empowerment. Members of the Ucayali Regional government expressed interest in documenting progress toward their zero‐deforestation land stewardship plan (described above). Funding for Alianza Cacao, CIAT, and Conservación Amazónicacame from the U.S. Agency for International Development (USAID). USAID emphasizes documentation of formal collaboration such as Memoranda Of Understanding. The scientists involved in the project (both from the United States and Peru) named that producing a peer‐reviewed publication was important for them. The Applied Earth Science SERVIR Program within the National Aeronautics and Space Administration (NASA) encourages funded scientists to orient around co‐produced project objectives rather than publication as an end‐goal. As long as the project involved using NASA earth observations to address local needs, SERVIR program requirements would be met. However, this encouragement did not alter the institutional career advancement criteria already in place within NASA Earth Science Division, which highly values peer‐review publication. In this way, the funding sources and institutional contexts shaped the unique motivation of each collaborator. Future work could involve working with our respective funding institutions to more closely align our incentives with those of our collaborating institutions.

## 5 CONCLUSION

We investigated the structural characteristics of agroforestry cacao, monocrop cacao, monocrop oil palm, and regenerating forest classes in Ucayali, Peru using a co‐production approach. In contrast to some of our hypotheses, results showed that secondary forests exhibited a similar vertical profile to mature forests and had significantly lower relative height ratios compared mature forest (*p* < .0001). This suggests numerous opportunities for future investigations of secondary versus mature forests in studies that controls for secondary forest age (rather than forest stage). While the use of lidar remote sensing for characterizing canopy structure of successional forest dynamics is increasingly common (Castillo‐Núñez et al., 2011; Clark et al., 2021; de Almeida et al., 2020; Feldpausch et al., 2005; Moran et al., 2018; Palace et al., 2016; Peña‐Claros, 2003; Pimmasarn et al., 2020), to date these have not specifically focused on characterizing the ways and extent to which structural patterns can be associated with tropical forest successional dynamics at landscape scales. Our work contributes to filling this knowledge gap. Simultaneously, we highlight how advancement of applied science can be carried out with a lens of co‐production and sustainable land stewardship—a set of aims only possible to achieve via inclusive and transdisciplinary collaboration.

## AUTHOR CONTRIBUTIONS

**Savannah S. Cooley:** Conceptualization (equal); data curation (equal); formal analysis (lead); funding acquisition (supporting); investigation (equal); methodology (equal); software (lead); supervision (equal); visualization (equal); writing – original draft (lead); writing – review and editing (equal). **Naiara Pinto:** Conceptualization (equal); data curation (equal); funding acquisition (lead); methodology (equal); project administration (lead); supervision (equal). **Milagros Becerra:** Conceptualization (equal); data curation (equal); investigation (equal); project administration (equal); resources (equal); writing – original draft (equal). **Jorge Washington Vela Alvarado:** Conceptualization (equal); data curation (equal); methodology (equal); resources (equal); validation (equal). **Jocelyn C. Fahlen:** Data curation (equal); methodology (equal). **Ovidio Rivera:** Conceptualization (equal); data curation (equal); investigation (equal); resources (equal). **G. Andrew Fricker:** Data curation (supporting); investigation (supporting); writing – review and editing (supporting). **Augusto Rafael De Los Ríos Dantas:** Conceptualization (equal); data curation (equal); methodology (equal); resources (equal). **Naikoa Aguilar‐Amuchastegui:** Conceptualization (equal); methodology (equal). **Yunuen Reygadas:** Data curation (equal); writing – review and editing (equal). **Julie Gan:** Visualization (equal). **Ruth DeFries:** Conceptualization (equal); resources (equal). **Duncan N. L. Menge:** Methodology (equal); resources (equal); supervision (equal); writing – review and editing (lead).

## CONFLICT OF INTEREST STATEMENT

The authors have no conflict of interest to declare.

### OPEN RESEARCH BADGES

This article has earned an Open Data badge for making publicly available the digitally‐shareable data necessary to reproduce the reported results. The data is available at https://doi.org/10.22002/D1.2318, https://lpdaac.usgs.gov/products/gedi02_av001/, and https://github.com/savcooley/ch1.GEDI_L2A_inR.git.

## Supporting information


Appendix S1.

## Data Availability

All data from this study are publicly available. Digitized polygons for each vegetation class are published on CaltechDATA repository: https://doi.org/10.22002/D1.2318. GEDI Level 2A data are accessible on NASA/USGS Land Processes Distributed Active Archive Center (LPDAAC): https://lpdaac.usgs.gov/products/gedi02_av001/. *Code availability*: The GEDI Level 2A processing code developed from this study is publicly available on GitHub: https://github.com/savcooley/ch1.GEDI_L2A_inR.git. We also created a technical document along with a training that provides step‐by‐step explanations of how to use GEDI Level 2A data for tropical ecosystem applications available in English and Spanish (also in same GitHub project).
