# Supplementary material for: Combining spaceborne lidar from the Global Ecosystem Dynamics Investigation with local knowledge for monitoring fragmented tropical landscapes: A case study in the forest–agriculture interface of Ucayali, Peru
Source: Ecol Evol. 2024 Aug 6;14(8):e70116. doi: 10.1002/ece3.70116 (PMC11303661; doi:10.1002/ece3.70116)
Supplement: Supplementary file 1 — Appendix S1. [file ECE3-14-e70116-s001.docx]

**Appendix**

# ***SI: Testing the impact of canopy height on RH50/RH95***

**Table S1.** (A) Linear regression model consisting of RH50/RH95 as the dependent variable with vegetation class and canopy height (as well as confounding factors of elevation and spatial coordinates) as the independent variables. To account for spatial autocorrelation, coordinates of points were included as interaction terms. Land cover coefficient estimates are relative to agroforestry cacao. The data represented have the negative height returns removed (i.e., the ground returns are not influencing the results). (B) Linear regression results from the original, untransformed data (i.e., the ground returns are still influencing the results).

A

B

| **Term** | **Estimate** | **Std.Error** | **Statistic** | **P-value** | **Signif.** |
| --- | --- | --- | --- | --- | --- |
| (Intercept) | -11.393 | 14.7753 | -0.7711 | 0.4407 |  |
| Canopy Height (RH95) | 0.0028 | 0.0002 | 11.3018 | 0.0000 | *** |
| Land Cover: Mature lowland forest | 0.1127 | 0.0147 | 7.6436 | 0.0000 | *** |
| Land Cover: Secondary lowland forest | 0.0842 | 0.015 | 5.6178 | 0.0000 | *** |
| Land Cover: Young vegetation regrowth | -0.0031 | 0.0192 | -0.1598 | 0.873 |  |
| Land Cover: Cacao - monocrop | 0.0119 | 0.0164 | 0.7234 | 0.4695 |  |
| Land Cover: Oil palm | 0.125 | 0.0139 | 9.0177 | 0.0000 | *** |
| elev_lowestmode | 0.0000 | 0.0000 | 0.5416 | 0.5881 |  |
| lat | -1.4243 | 1.7298 | -0.8234 | 0.4103 |  |
| lon | -0.1599 | 0.1967 | -0.8128 | 0.4164 |  |
| lat:lon | -0.0190 | 0.0230 | -0.8254 | 0.4092 |  |
|  |  |  |  |  |  |
|  |  |  |  |  |  |

| **Term** | **Estimate** | **Std.Error** | **Statistic** | **P-value** | **Signif.** |
| --- | --- | --- | --- | --- | --- |
| (Intercept) | -37.0649 | 22.1475 | -1.6735 | 0.0943 | * |
| Canopy Height (RH95) | 0.0073 | 0.0004 | 20.7714 | 0.0000 | *** |
| Land Cover: Mature lowland forest | 0.1688 | 0.0211 | 8.0057 | 0.0000 | *** |
| Land Cover: Secondary lowland forest | 0.1358 | 0.0214 | 6.3460 | 0.0000 | *** |
| Land Cover: Young vegetation regrowth | -0.0127 | 0.0273 | -0.4638 | 0.6428 |  |
| Land Cover: Cacao - monocrop | 0.0009 | 0.0233 | 0.0377 | 0.9699 |  |
| Land Cover: Oil palm | 0.2126 | 0.0198 | 10.7189 | 0.0000 | *** |
| elev_lowestmode | 0.0000 | 0.0000 | 0.1618 | 0.8715 |  |
| lat | -4.3974 | 2.5955 | -1.6942 | 0.0903 | * |
| lon | -0.4989 | 0.2947 | -1.6929 | 0.0906 | * |
| lat:lon | -0.0586 | 0.0345 | -1.6961 | 0.0899 | * |
|  |  |  |  |  |  |
| Signif. codes: ‘***’ <0.001 ‘**’ <0.01 ‘*’ <0.1 | |  |  |  |  |
| Residual standard error: 0.1301 on 3709 degrees of freedom |  |  |  |  |  |
| Multiple R-squared: 0.3261, Adjusted R-squared: 0.3243 | | |  |  |  |
| F-statistic: 179.5 on 10 and 3709 DF | | |  |  |  |
|  |  |  |  |  |  |

**
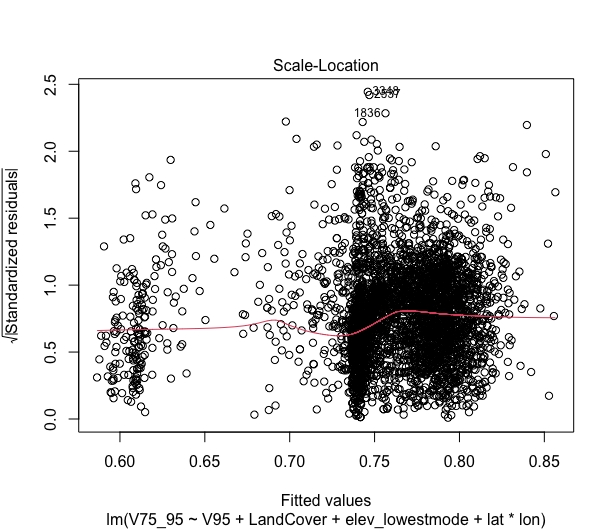
**

**Figure S1.** Scale-location plot (also known as the spread-location plot) showing homogeneity of variance in the residual errors and supporting the assumption of homoscedasticity. The mean variability (variances) of the residual points slightly increases with the value of the fitted outcome variable and then slightly decreases again. However, the magnitude of this change is small, remaining between 0.5 and 1 of the square root of the absolute value of the standardized residuals.


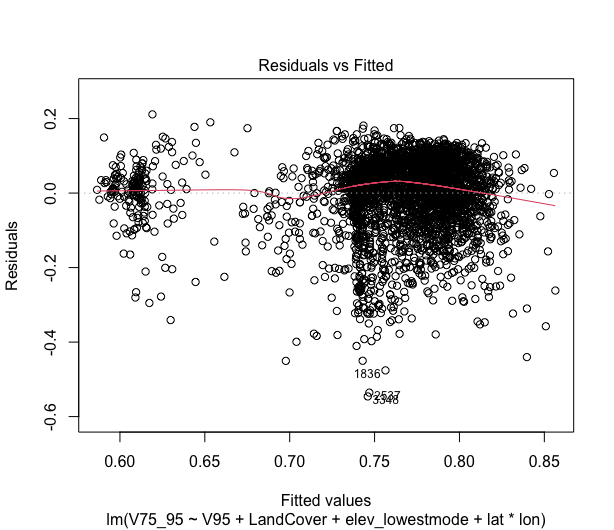


**Figures S2**. The residual plot shows no fitted pattern (the red line is approximately horizontal at zero). This supports the assumption of linearity.


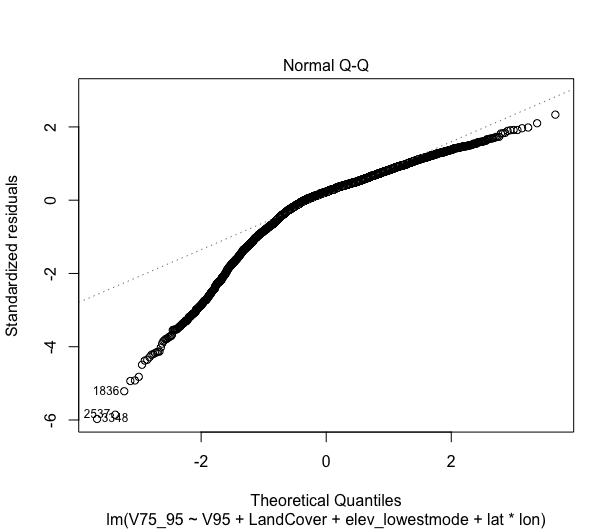


**Figures S3**. The normal probability plot of residuals (QQ plot) does not follow a straight line. The assumption of normality of residuals was therefore not met (also confirmed by the Shapiro-Wilk test W=0.86013, p-value <0.0001).

**Figures S4**. Alternative version of Figure 3: error bars show 1 standard deviation instead of 1 standard error. Mean and standard error of relative height (RH) metrics (i.e., cumulative energy levels) for (A, C) tropical lowland forest regeneration classes including mature forest, secondary and young vegetation (‘purma’); and (B, D) agricultural classes including oil palm plantations (all monocrop) and cacao plantations (both monocrop and agroforestry). Negative height values represent the ground returns in each observation, which arises due to low canopy cover. Figures B and D show results from removing the negative height returns and interpolating each observation from the remaining above-ground observations.

**Figures S5**. Alternative version of Figure 4: results from original data with no cumulative energy offset. RH metrics and ratios across vegetation class types: (A) mean canopy height (RH95), (B) the height of median energy (HOME; RH50), (C) the ratio of HOME to canopy height (RH95) (RH50/RH95) and (D) the ratio of RH75 to canopy height (RH95) (RH75/RH95). All data reported in this graph have been left untransformed (i.e., the ground returns influence the results).

# ***SI: What we learned about co-production and participatory land cover mapping***

While our co-development approach was based on the findings of land cover mapping initiatives in the Amazon as well as existing citizen science-based land cover mapping literature, our lessons learned could apply to many other land cover mapping projects globally. In particular, we highlight 3 areas of learning, with a special focus on gender and women’s empowerment. These include: (1) Create a shared vision, outcomes and expectations for the collaborative project; (2) Co-develop joint concepts and land cover keys; and (3) Cultivate a culture of two-way knowledge exchange.

## 2.1. Creating shared, mutually valued vision and outcomes

Establishing shared and mutually valued vision, research question and outcomes for the project from the perspective of both the research and non-scientist participants forms the foundation for effective co-developed, collective action participatory mapping (Turnhout et al., 2020). This is particularly important in the context of engaging women in citizen science efforts, who often face additional barriers to participation in citizen science activities (Asingizwe et al., 2020; Kalpazidou Schmidt & Cacace, 2017; Paleco et al., 2021). Creating a shared vision for the collaborative project also helps address a challenge that often arises in citizen science for earth observation projects: building and maintaining citizen engagement (Fritz et al., 2017).

In our project, shared vision and ownership coalesced around the desire to map agricultural and secondary vegetation in the region. From the researchers’ perspective, the desired outcomes of our project involved reconciling detailed local knowledge of the vegetation and ecosystems with the capabilities and limitations of the lidar data intended to be used for the generation of vegetation signature libraries. From the perspectives of the local collaborators, broad recognition of the need to map agricultural and unmanaged ecosystems led to the support of exploring possible approaches with active remote sensing capabilities.

## 2.2. Co-development of joint concepts and land cover keys

The development and refinement of the vegetation categories repeatedly underscored the importance of defining joint concepts and land cover keys. When communicating, both the researchers as well as the local experts did their best to avoid the use of technical jargon to reduce barriers to understanding and avoid exclusionary practices (Rakedzon et al., 2017). This was particularly important due to the language and cultural barriers, which brought additional complexity to the communication process. The use of the term “purma” represents an example of local terminology that we found to be important for the team to dedicate numerous conversations towards clearly defining (Table 2; Malleux 1986; Dourojeanni 1990; informal interview, March 2021).

An even broader and more complex concept that our project dedicated effort to exploring was the concept of women empowerment in science. In particular, our cacao mapping activity solely worked with women farmers, a choice rooted in the recognition of the need to foster female leadership in science and land stewardship. We sought to celebrate the vast knowledge of cacao cultivation that each woman farmer possessed via directly incorporating what the farmers shared into the land cover key definitions. The women farmers led the data collection and capturing of photos, all of which contributed to the final land cover key.

A more technical example of this best practice can also be provided in the cacao mapping activity. Our team created an initial presentation that introduced basic concepts of map making and remote sensing such as: “spatial coordinates”, “extrapolation”, “upscaling”, “land cover” vs. “land use”, “representativeness”, and “remote sensing signatures”. Defining these terms early on in the project development allowed the women farmers to fully conceptualize the proposed project in its entirety and clear up any misunderstandings. For instance, during the discussion about the differences between “land cover” vs. “land use”, the SERVIR-Amazonia team emphasized that the land cover maps are not meant to locate individual land owners, which was a common point of confusion and concern.

## 2.3. Cultivating a culture of two-way knowledge exchange

The cultivation of two-way knowledge exchange between all collaborators emerged as a priority starting from the initial discussions for the activity. The U.S.-based scientists recognized the need to learn from local knowledge of the vegetation and ecosystems in order to effectively categorize the landscape typologies planned to form the foundation for the subsequent active remote sensing library development. The local collaborators, in turn, wanted to learn more about spaceborne lidar technology and its applications for land cover mapping. This provided fertile ground for the shared understanding that we all have something to teach and we all have something to learn.

In addition to contributing to the land cover key definitions and collecting data, the women farmers witnessed their knowledge of cocoa cultivation practices inform the study’s hypotheses – another core component of the scientific method. Knowledge from the female farmers prompted the expectation that we would see high structural variability not only among the cacao agroforestry plantations, but in the monocrop plantations as well. This full participation provided a direct affirmation of the knowledge and wisdom each woman farmer shared.
